# Quality of Life in Older Adults After Major Cancer Surgery: The GOSAFE International Study

**DOI:** 10.1093/jnci/djac071

**Published:** 2022-04-08

**Authors:** Isacco Montroni, Giampaolo Ugolini, Nicole M Saur, Siri Rostoft, Antonino Spinelli, Barbara L Van Leeuwen, Nicola De Liguori Carino, Federico Ghignone, Michael T Jaklitsch, Ponnandai Somasundar, Anna Garutti, Chiara Zingaretti, Flavia Foca, Bernadette Vertogen, Oriana Nanni, Steven D Wexner, Riccardo A Audisio, the SIOG Surgical Task Force/ESSO GOSAFE Study Group, Giovanni Taffurelli, Davide Zattoni, Paola Tramelli, Giacomo Sermonesi, Giorgio Ercolani, Francesca Tauceri, Barbara Perenze, Daniela Di Pietrantonio, Mariateresa Mirarchi, Gianluca Garulli, Vincenzo Alagna, Andrea Lucchi, Basilio Pirrera, Francesco Monari, Luigi Conti, Patrizio Capelli, Andrea Romboli, Gerardo Palmieri, Filippo Banchini, Francesca Di Candido, Michele Carvello, Matteo Sacchi, Francesca De Lucia, Caterina Foppa, Luigi Marano, Alessandro Spaziani, Giampaolo Castagnoli, Alberto Bartoli, Laura Frain, Sam W Fox, Kristin Cardin, Luis E De Leon, Mario Trompetto, Gaetano Gallo, Alberto Realis Luc, Giuseppe Clerico, Giuseppe Sammarco, Raffaele De Luca, Michele Simone, Rocco Lomonaco, Michael Fejka, Joshua I S Bleier, Matthijs Plas, Hanneke van der Wal-Huisman, Andrea Costanzi, Giulio Mari, Dario Maggioni, Roberta Pellegrino, Roberta Pellegrino, Pietro Ascheri, Jakub Kenig, Kinga Szabat, Stefano Scabini, Davide Pertile, Lorenzo Epis, Andrea Massobrio, Domenico Soriero, Arild Nesbakken, Ingeborg Flåten Backe, Mariann Lønn, Giovanni Ferrari, Michele Mazzola, Carmelo Magistro, Pietro Achilli, Alessandro Giani, Orestis Ioannidis, Lydia Loutzidou, Konstantinos Galanos-Demiris, Genoveffa Balducci, Barbara Frezza, Alessio Lucarini, Claudia Santos, Diogo Cardoso, Isabela Gil, Vasco Cardoso, Lisa Cooper, Baha Siam, Yochai Levy, Baruch Brenner, Hanoch Kashtan, Valerio Belgrano, Franco Decian, Beatrice Palermo, Roberto Eggenhöffner, Manuela Albertelli, Luis Sánchez-Guillén, Antonio Arroyo, Francisco López-Rodríguez, Sandra Lario, Cristina Lillo, Minas Baltatzis, Anthony K C Chan, Ajith K Siriwardena, Giovanna Da Silva

**Affiliations:** Colorectal surgery Unit, Ospedale “per gli Infermi”, AUSL Romagna, Faenza, Italy; Colorectal surgery Unit, Ospedale “per gli Infermi”, AUSL Romagna, Faenza, Italy; Perelman School of Medicine, Department of Surgery, Division of Colon and Rectal Surgery, University of Pennsylvania, Philadelphia, PA, USA; Department of Geriatric Medicine, Oslo University Hospital and Institute of Clinical Medicine, University of Oslo, Oslo, Norway; Department of Biomedical Sciences, Humanitas University, Milan, Italy; IRCCS Humanitas Research Hospital, Rozzano, Milan, Italy; Department of Surgical Oncology, University Medical Center Groningen, University of Groningen, Groningen, the Netherlands; HPB Unit, Manchester Royal Infirmary, University of Manchester, Manchester, UK; Colorectal surgery Unit, Ospedale “per gli Infermi”, AUSL Romagna, Faenza, Italy; Division of Thoracic Surgery and Division of Aging, Brigham and Women’s Hospital, Boston, MA, USA; Department of Surgery, Roger Williams Medical Center, Boston University, Providence, RI, USA; Colorectal surgery Unit, Ospedale “per gli Infermi”, AUSL Romagna, Faenza, Italy; Unit of Biostatistics and Clinical Trials, IRCCS Istituto Romagnolo per lo Studio dei Tumori (IRST) “Dino Amadori”, Meldola, Italy; Unit of Biostatistics and Clinical Trials, IRCCS Istituto Romagnolo per lo Studio dei Tumori (IRST) “Dino Amadori”, Meldola, Italy; Unit of Biostatistics and Clinical Trials, IRCCS Istituto Romagnolo per lo Studio dei Tumori (IRST) “Dino Amadori”, Meldola, Italy; Unit of Biostatistics and Clinical Trials, IRCCS Istituto Romagnolo per lo Studio dei Tumori (IRST) “Dino Amadori”, Meldola, Italy; Department of Colorectal Surgery, Cleveland Clinic Florida, Weston, FL, USA; Department of Surgery, Institute of Clinical Sciences, Sahlgrenska University Hospital, Göteborg, Sweden

## Abstract

**Background:**

Accurate quality of life (QoL) data and functional results after cancer surgery are lacking for older patients. The international, multicenter Geriatric Oncology Surgical Assessment and Functional rEcovery after Surgery (GOSAFE) Study compares QoL before and after surgery and identifies predictors of decline in QoL.

**Methods:**

GOSAFE prospectively collected data before and after major elective cancer surgery on older adults (≥70 years). Frailty assessment was performed and postoperative outcomes recorded (30, 90, and 180 days postoperatively) together with QoL data by means of the three-level version of the EuroQol five-dimensional questionnaire (EQ-5D-3L), including 2 components: an index (range = 0-1) generated by 5 domains (mobility, self-care, ability to perform the usual activities, pain or discomfort, anxiety or depression) and a visual analog scale.

**Results:**

Data from 26 centers were collected (February 2017-March 2019). Complete data were available for 942/1005 consecutive patients (94.0%): 492 male (52.2%), median age 78 years (range = 70-95 years), and primary tumor was colorectal in 67.8%. A total 61.2% of all surgeries were via a minimally invasive approach. The 30-, 90-, and 180-day mortality was 3.7%, 6.3%, and 9%, respectively. At 30 and 180 days, postoperative morbidity was 39.2% and 52.4%, respectively, and Clavien-Dindo III-IV complications were 13.5% and 18.7%, respectively. The mean EQ-5D-3L index was similar before vs 3 months but improved at 6 months (0.79 vs 0.82; *P* < .001). Domains showing improvement were pain and anxiety or depression. A Flemish Triage Risk Screening Tool score greater than or equal to 2 (odds ratio [OR] = 1.58, 95% confidence interval [CI] = 1.13 to 2.21, P = .007), palliative surgery (OR = 2.14, 95% CI = 1.01 to 4.52, *P* = .046), postoperative complications (OR = 1.95, 95% CI = 1.19 to 3.18, *P* = .007) correlated with worsening QoL.

**Conclusions:**

GOSAFE shows that older adults’ preoperative QoL is preserved 3 months after cancer surgery, independent of their age. Frailty screening tools, patient-reported outcomes, and goals-of-care discussions can guide decisions to pursue surgery and direct patients’ expectations.

The number of older cancer patients is rapidly increasing (1), but the optimal surgical care and outcomes remain underinvestigated. Despite acknowledging that this age group is unique and heterogeneous, information on the implications of surgery on individual patients is often limited (1-3). Knowledge comes from retrospective databases or institutional experiences (4). Older and/or frail patients are often excluded from large and randomized control trials (4,5).

Retrospective analysis carries well-known biases and prevents researchers from answering relevant, real-world questions such as “How is my life going to change after surgery?” and “Will I be functional again after treatment?” (6). Cancer has become a leading cause of death in older patients, and treatment plans may affect the quality of life (QoL) (7). The objective is to identify the most appropriate treatment based on the patient’s cancer, function, and goals.

The literature is unable to answer these fundamental queries because QoL, functional recovery (FR), and other patient-reported outcomes (PROs) are missing from prospective studies, and older patients were often excluded from relevant investigations (8).

The goal of the Geriatric Oncology Surgical Assessment and Functional rEcovery after Surgery (GOSAFE) study was to obtain prospective data on the variation of both QoL and FR after major cancer surgery. The GOSAFE study was promoted by a multidisciplinary group supported by the European Society of Surgical Oncology (ESSO) and the Surgical Task Force of the International Society of Geriatric Oncology (SIOG) to investigate how the association between frailty assessment and PROs will shift care to personalized treatment and aim to improve clinical and functional outcomes. We report on the primary outcome: QoL derangement after surgery and factors predicting worsening in QoL. Secondary outcomes, postoperative morbidity or mortality, and factors predicting postoperative mortality are also reported.

## Methods

The GOSAFE study was a multicenter, international, prospective observational cohort study carried out at 26 hospitals worldwide (Supplementary Table 1, available online). This study was approved by each center’s institutional review board and ethics committee. The study was registered at https://clinicaltrials.gov/ (identifier: NCT03299270). The study protocol is available in the Supplementary Material (available online).

### Inclusion Criteria

All consecutive patients aged 70 years and older undergoing elective major surgery with curative or palliative intent for a solid malignancy were eligible. Primary procedures (ie, any resection for any cancer via any operative approach) were included. The goal was to obtain information about real-life practices in the surgical care of older patients; therefore, cognitive impairment was not considered an exclusion criterion. Informed consent was obtained by the appropriate health-care proxy.

### Exclusion Criteria

Patients undergoing emergent or urgent surgery or planned hospital stay less than 48 hours were excluded. Centers failing to provide at least 20 consecutive patients were excluded.

The heterogeneity in the study population was instrumental to a real-world investigation, aiming to define possible risk factors predicting variations in QoL, FR, and symptoms.

The primary outcome of the study was the comparison of QoL data before and after surgery (3 and 6 months). The use of the the three-level version of the EuroQol five-dimensional questionnaire (EQ-5D-3L) in the surgical population was previously described and internationally validated; it includes 2 components: an index and a visual analog scale (VAS) score (Supplementary Figure 1) (9,10). The index is based on patients’ satisfaction in 5 domains (5D: mobility, self-care, ability to perform usual activities, pain or discomfort, anxiety or depression) across 3 levels (3L: no problem, some problems, extremely problematic). Based on the scoring of all 5 domains and through an algorithm that converts individual patients' answers into a global index based on the country of origin, a score is calculated ranging from 0 (poorest QoL) to 1 (best possible QoL). The value set generated from the European population (EQ) was used as the reference cohort (11). The VAS score is generated by asking the patient how they ranked their QoL compared with their peers on a scale from 0 to 100.

There are several advantages of the EQ-5D-3L, including ease and speed of implementation in a busy surgical practice. In addition, the EQ-5D-3L had been validated in numerous surgical studies (12,13) and for use in patients with cognitive disorders (14).

Secondary outcomes were 1) to detect short- and long-term postoperative morbidity and mortality, and 2) to detect any association between risk factors (including data resulting from the frailty assessment tools) and QoL. Because morbidity and mortality are linked with QoL, predictors of 90-day mortality were assessed and morbidity was recorded (scored according to the Clavien-Dindo classification complication grade [CD] classification) (15). The CD classification only accounts for the most severe adverse event for each patient; however, all complications were recorded during the study period for each patient because, especially in older adults, complications are typically cumulative.

The baseline evaluation and postoperative follow-up assessments require approximately 20 minutes to complete and were carried out by health-care providers in the outpatient clinic as part of routine preoperative evaluation (16). No formal prehabilitation protocol was required, and optimization was performed for individual patients based on the center’s standard practice. Postoperative follow-up visits at 90 and 180 days allowed measuring of QoL and functional status. When a routine office visit was not planned, an ad-hoc in-person appointment or telephone interview was scheduled.

The selection of frailty screening tools was based on the level of evidence from the literature and previous experience from the research consortium (17,18). The Eastern Collaborative Oncology Group Performance Status (ECOG PS) (19), Katz Activities of Daily Living (20), the 5-item Flemish version of the Triage Risk Screening Tool (fTRST) (21,22), the Timed Up and Go (TUG) test (23), the Geriatric 8 (G8) (24), the Nutritional Risk Screening (25), American Society of Anesthesiologists (ASA) score (26), and the Charlson Age Comorbidity Index (CACI) (27). Specifics about frailty screening tests and perioperative assessment were previously reported (Supplementary Table 2, available online) (16).

Online inspections were conducted by the research coordinators (Z.C. and F.F.) to maintain the highest possible data quality. STrengthening the Reporting of OBservational studies in Epidemiology reporting standards for cohort studies were entirely followed.

## Statistical Analysis

Continuous variables were reported as median (minimum-maximum values) or mean (SD). Categorical variables were reported by means of frequencies. McNemar's test was used to evaluate change in the domains of EQ-5D-3L from baseline to 3 or 6 months after surgery.

Primary outcome analysis for missing data was performed using a complete case analysis.

Patients who died before the 3-month evaluation were excluded from the QoL data analysis (28). These cases were considered in the mortality data analysis and reported in the Results section.

Logistic regression analyses investigating the association of variables with the outcomes of death and worsened EQ-5D-3L index (yes or no) at 3 or 6 months after surgery were performed. Crude and adjusted odds ratios (ORs) with 95% confidence intervals (CIs) were reported. A *P* value of less than .05 was considered statistically significant.

Stepwise regression models starting with no predictor were carried out, and final models for mortality and QoL were chosen according to a criteria based on statistical appropriateness to contain partial overlapping of domains and items from the frailty screening tools. Association among frailty screening variables was explored through a stratified analysis, and test of homogeneity was carried out. 

Akaike Information Criteria (AIC) and Bayesian Information Criteria (BIC) were used for model comparison considering the fit and complexity of the models. Models with the lower BIC and AIC were superior for reporting the relationship between outcomes and risk factors. During stepwise process, when variables were found to be associated, the ones generating the model with best BIC and AIC were selected.

The VAS score components of the EQ-5D-3L and the EQ-5D-3L index were considered continuous variables, and an analysis of variance for repeated measures was performed comparing preoperative vs 3-month vs 6-month results. Bonferroni’s correction was used for post hoc comparisons. Linear mixed effects models, which are robust to individual changes, were used to analyze EQ-5D VAS score or EQ-5D index as dependent variable focusing on the main effect of time (baseline, evaluation at 3 months, and evaluation at 6 months) accounting for patient intra-variability. All statistical analyses were carried out using Stata/MP 15.0 for Windows (Stata-Corp LP, College Station, TX, USA).

## Results

**Figure 1.**
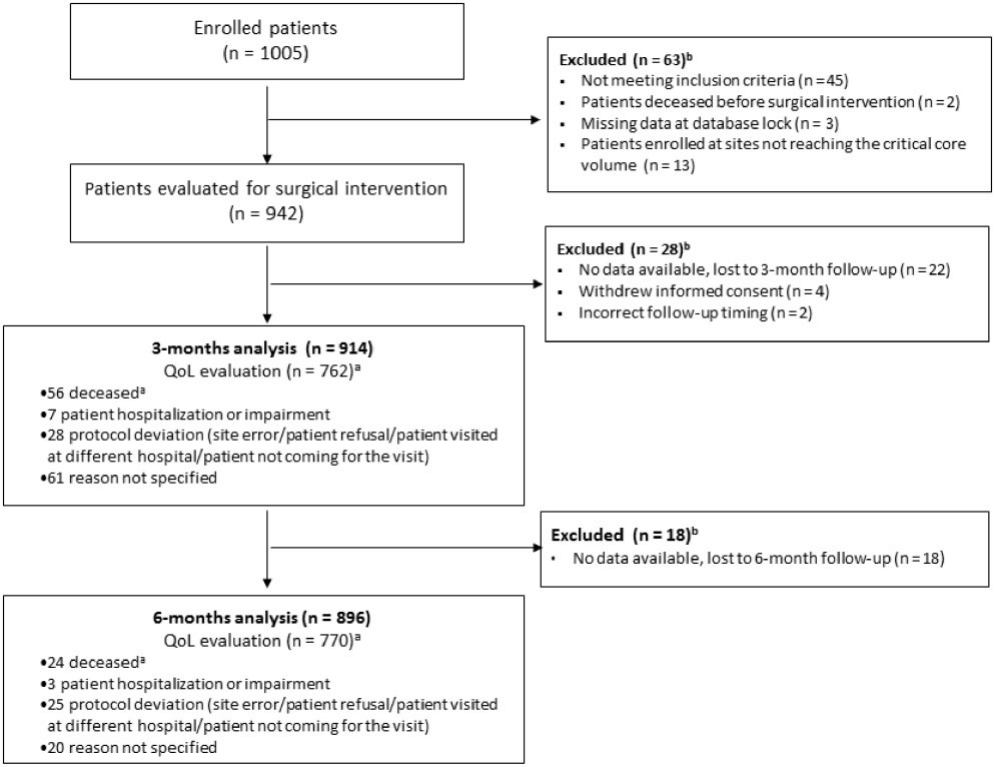
Geriatric Oncology Surgical Assessment and Functional rEcovery after Surgery (GOSAFE) study flow diagram. ^a^missing for quality of life (QoL) analysis only. ^b^patients with no data available.

Data from 26 centers were prospectively collected from February 2017 to March 2019 on 942 out of 1005 (94.0%) initially enrolled patients. Three patients had incomplete baseline data, 47 did not meet the inclusion criteria, and 2 patients died before surgery. Thirteen patients enrolled from centers that did not achieve the critical core volume were also excluded (Figure 1).

There were 492 men (52.2%), and the median age was 78 years (range = 70-95 years). Primary tumor was colorectal cancer in 67.8% patients (633 of 942); 61.2% (572 of 942) of surgeries were performed via minimally invasive approach. A total 51.9% patients lived alone, 47.3% were at home with family or caregivers, and only 8 patients (0.8%) resided in residential care facilities before surgery (Table 1). A procedure-specific breakdown is included in Supplementary Table 3 (available online).

**Table 1.** Demographic data and baseline frailty screening

Variable	Overall (n = 942)
	No. (%)
Sex	
Male	492 (52.2)
Female	450 (47.8)
Median age, y (range)	78 (70-95)
Age, y	
70–74	299 (31.7)
75–79	295 (31.3)
80–84	233 (24.7)
≥85	115 (12.3)
Living situation	
Home independent	489 (51.9)
Home with family or caregiver	445 (47.3)
Residential care	8 (0.8)
Medication use	
None	57 (5.9)
Median no. of drugs (range)	4 (1-28)
History of falls 6 mo before operation	92 (9.8)
Previous delirium	52 (5.5)
Smoking habits	
Yes	78 (8.3)
No (former)	409 (43.5)
No (never)	454 (48.2)
Missing	1
Cancer site	
Endocrine	8 (0.9)
Upper GI	105 (11.2)
Lower GI	633 (67.8)
HBP	104 (11.1)
Soft tissue or bone	14 (1.5)
Thoracic	36 (3.9)
Genito-urinary	10 (1.1)
Gyn	3 (0.3)
Other	21 (2.2)
Unknown	8
Type of surgery	
Curative	885 (94.8)
Palliative	49 (5.2)
Unknown	8
Surgical approach	
Open	354 (37.9)
Minimally invasive	536 (57.3)
Robotic	36 (3.9)
Other	8 (0.9)
Missing	8
G8 score	
G8 ≤ 14^a^	648 (68.9)
G8 > 14	293 (31.1)
Missing	1
fTRST	
0	233 (24.8)
1	363 (38.6)
≥2^a^	345 (36.6)
Missing	1
ADL SCORE	
<5^a^	76 (8.1)
≥5	860 (91.9)
Missing	6
MiniCog Total score	
0–2^a^	193 (20.8)
3–5	734 (79.2)
Missing	15
ASA score	
1–2	467 (50.7)
3–4^a^	455 (49.3)
Missing	20
PS ECOG	
ECOG 0	520 (55.5)
ECOG 1	274 (29.3)
ECOG ≥ 2^a^	152 (15.2)
Missing	6
Charlson Age Comorbidity Index	
3–6	610 (64.8)
≥7^a^	332 (35.2)
Timed Up and Go	
≤ 20 s	790 (94.6)
>20 s^a^	45 (5.4)
Missing	107
Nutritional status score	
Normal	607 (65.5)
Mildly impaired	230 (24.8)
Moderately impaired^a^	68 (7.3)
Severely impaired	22 (2.4)
Missing	15
Laboratory tests, median (range)	
Albumin, g/L	40 (20-70)
Hgb, g/dL	12.3 (6.10-17.4)
Creatinine, mg/dL	0.9 (0.15-11.3)

^a^ Indicates abnormal values. Please see Supplementary Table 2 (available online) for frailty screening tests and threshold. ADL = activities of daily living; ASA = American Society of Anesthesiologists; PS ECOG = Performance Status Eastern Cooperative Oncology Group; fTRST = Flemish version of the Triage Risk Screening Tool ; G8 = Geriatric 8; GI = gastrointestinal; HBP = pancreas, duodenum, liver, biliary tree; Hgb = hemoglobin; Other = intrabdominal sarcoma, spleen, pelvic recurrence of rectal adenocarcinoma, colon cancer with gastric infiltration.

The number of patients with positive indicators of frailty varied extensively by the frailty screening tool used from 5.4% to 68.9% of the cohort (Table 1).

QoL data were available for 942 patients at baseline, 762 patients at 3 months, and 770 at 6 months (69 patients missed the 3-month visit but followed-up at 6 months for QoL analysis). Eighty-nine (89 of 942, 9.4%) and 45 (45 of 942, 4.8%) patients were lost to follow-up or follow-up was not correctly performed (ie, QoL test not administered during follow-up visits) at 3 and 6 months, respectively (Figure 1).

**Figure 2.**
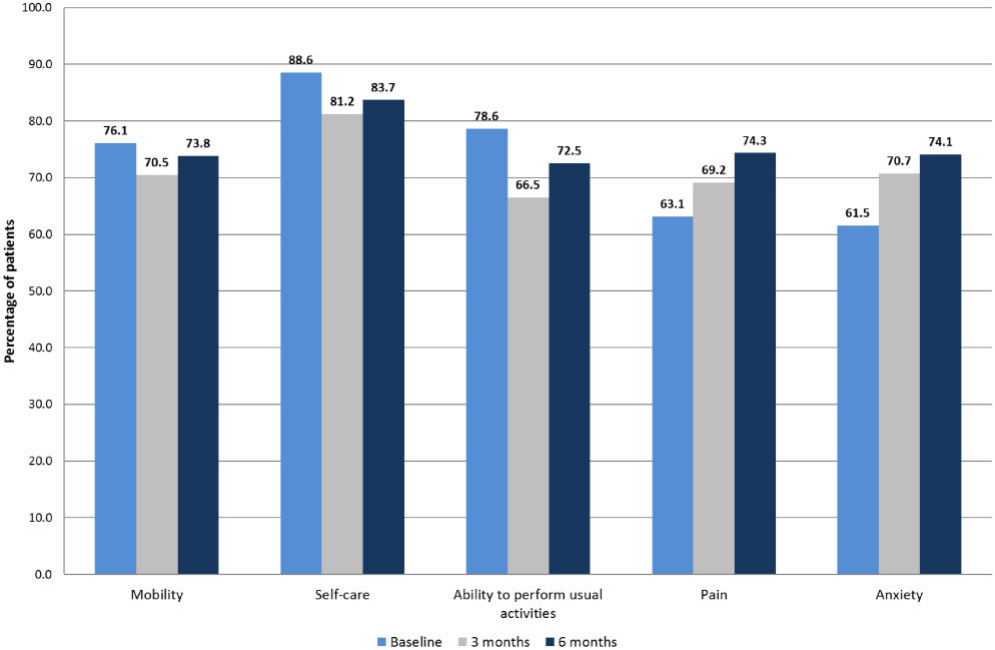
Percentage of patients without deficits at baseline, 3 months, and 6 months for the 5 EQ-5D-3L domains.

Data showed that at 3 months after surgery, most patients reported stable QoL according to the EQ-5D-3L compared with their preoperative status (Figure 2). Repeated-measures analysis based on the EQ-5D index showed an improvement over time (*P* < .001). Based on the EQ-5D index, QoL was improved 6 months after surgery. The mean EQ-5D-3L index was equivalent before and at the 3-month mark (baseline value = 0.79, SD = 0.21 vs 3-month value = 0.79, SD = 0.23, *P* > .99), and it was statistically significantly improved 6 months after surgery (baseline value = 0.79, SD = 0.20 vs 6-month value = 0.82, SD = 0.22, *P* = .01). The linear mixed effect model, accounting for variability for individual patients, showed an effect of time over EQ-5D index (beta coefficient = 0.013, *P* = .001).

Similar results were recorded when analyzing the visual component of the EQ-5D-3L. The mean VAS score was 69.9 (SD = 18.3) before surgery vs 71.3 (SD = 19.4) and 73.3 (SD = 16.1) at 3 and 6 months. The analysis of variance of the VAS scale on 3 time points was statistically significant (*P* < .001), and the post hoc comparison revealed a statistically non-significant variation between the preoperative and 3-month evaluations (*P* = .06), whereas at 6 months, the variation was statistically different (*P* < .001). The linear mixed effect model showed an effect of time over EQ-5D VAS (beta coefficient = 1.381, *P* < .001).

Figure 2 demonstrates the fluctuation of EQ-5D-3L domains. After 3 and 6 months, both pain and anxiety or depression improved, with 69.2% and 74.3%, respectively, and 70.7% and 74.1% of patients, respectively, reporting no issues at 3 or 6 months (compared with 63.1% and 61.5%, respectively, before surgery, *P* < .001). Conversely, analysis of mobility, self-care, and ability to perform usual activities showed a lower level of satisfaction after surgery (76.1%, 88.6%, and 78.6%, respectively, before surgery vs 73.8%, 83.7%, and 72.5%, respectively, at 6 months with *P* = .03, <.001, and .001, respectively).

Logistic regression analysis was performed to identify risk factors for worsening QoL at 3 and 6 months. Patients were dichotomized into 2 groups: 1) patients who had a higher or equal preoperative EQ-5D-3L index than postoperative, and 2) patients who had a lower (worse) index preoperatively than postoperative. Table 2 reports the univariate and multivariable analysis. At 3 months, postoperative complications (OR = 1.67, 95% CI = 1.17 to 2.38, *P* = .004 for CD I-II; OR = 2.20, 95% CI = 1.34 to 3.61, *P* = .002 for CD III-IV) correlated with worsening QoL. A trend was also noted in the 3-month analysis for history of delirium (OR = 1.94, 95% CI = 0.99 to 3.76, *P* = .05) and for fTRST ≥ 2 (OR = 1.37, 95% CI = 0.98 to 1.92, *P* = .06). This trend was confirmed in the 6-month analysis for fTRST ≥ 2 (OR = 1.58, 95% CI = 1.13 to 2.21, *P* = .007), postoperative CD III-IV complications (OR = 1.95, 95% CI = 1.19 to 3.18, *P* = .007), and palliative surgery (OR = 2.14, 95% CI = 1.01 to 4.52, *P* = .046) that are all related with reduced QoL. Age 80 years and older, ASA score, and disease location (other sites vs lower gastrointestinal) were not associated with decreased QoL.

**Table 2.** Univariate and multivariable model showing association with perioperative parameters and likelihood to have worse QoL as measured by the EQ-5D-3L index at 3 and 6 months after surgery^a^

	3-month QoL				6-month QoL			
Baseline covariates	OR for univariate model (95% CI)	*P*	OR for multivariable model (95% CI)	*P*	OR for univariate model (95% CI)	*P*	OR for multivariable model (95% CI)	*P*
Age								
≥80 y vs <80 y	0.95 (0.69 to 1.30)	.76			1.06 (0.77 to 1.44)	.73		
ASA score								
3–4 vs 1–2	1.21 (0.89 to 1.64)	.21			1.02 (0.75 to 1.39)	.87		
CACI								
≥7 vs 3–6	1.27 (0.92 to 1.76)	.13			1.26 (0.91 to 1.74)	.16		
fTRST								
≥2 vs <2	1.66 (1.21 to 2.26)	.002	1.37 (0.98 to 1.92)	.06	1.79 (1.30 to 2.46)	<.001	1.58 (1.13 to 2.21)	.007
G8 score								
≤14 vs >14	1.49 (1.06 to 2.09)	.020	1.34 (0.93 to 1.91)		1.55 (1.10 to 2.18)	.01	1.38 (0.96 to 1.99)	.08
PS ECOG								
≥2 vs 0 to 1	1.40 (0.92 to 2.13)	.11			1.42 (0.92 to 2.19)	.11		
NRS								
Mildly impaired vs normal	0.78 (0.54 to 1.12)	.18			1.00 (0.70 to 1.44)	.96		
Mod + sev impaired vs normal	0.66 (0.38 to 1.16)	.15			1.14 (0.68 to 1.95)	.60		
TUG								
>20 vs ≤20	1.85 (0.94 to 3.64)	.07			1.81 (0.91 to 3.60)	.09		
Surgical approach								
Open vs minimally invasive or robotic	1.31 (0.96 to 1.79)	.09			1.21 (0.88 to 1.65)	.23		
Type of surgery								
Palliative vs curative	1.75 (0.89 to 3.47)	.10			2.42 (1.17 to 4.98)	.02	2.14 (1.01 to 4.52)	.046
Previous delirium								
Yes vs no	2.61 (1.37 to 4.96)	.003	1.94 (0.99 to 3.76)	.05	1.55 (0.81 to 2.99)	.18		
Complication at surgery								
Only GI–GII vs none	1.85 (1.31 to 2.61)	<.001	1.67 (1.17 to 2.38)	.004	1.08 (0.75 to 1.56)	.66	0.99 (0.68 to 1.44)	.98
At least GIII–IV complication vs none	2.57 (1.59 to 4.18)	<.001	2.20 (1.34 to 3.61)	.002	2.23 (1.38 to 3.59)	.001	1.95 (1.19 to 3.18)	.007
Site of disease								
Lower GI vs other site of disease	1.37 (0.98 to 1.92)	.06	1.26 (0.89 to 1.79)	.18	1.41 (1.01 to 1.95)	.04	1.35 (0.95 to 1.89)	.08

^a^ ASA=American Society of Anesthesiologists; CACI = Charlson Age Comorbidity Index; CI = confidence interval; fTRST=Flemish version of the Triage Risk Screening Tool; GI = gastrointestinal; mod + sev = moderately and severely; NRS = nutritional risk screening; OR = odds ratio; PS ECOG=Performance Status Eastern Cooperative Oncology Group; QoL = quality of life; TUG = timed up and go; GI-GII = Grade I or Grade II (Clavier-Dindo classification); GIII-GIV = Grade III or Grade IV (Clavier-Dindo classification)

Association analysis between frailty screening variables that were statistically significant in the univariate analysis (fTRST, G8 scores) was investigated before the multivariable model due to multicollinearity: stratified analysis reported a similar odds ratio of being fTRST of at least 2 for patients with G8 of 14 or less and patients G8 greater than 14 as well as odds ratio of being G8 14 or less was similar for patients with a fTRST of at least 2 and patients with a fTRST less than 2 (*P*_homogeneity_ = .86 for both comparisons). Interaction among G8 and fTRST was not statistically significant (*P*_interaction_ = .85). These results confirm there was no evidence of interaction.

Supplementary analysis was performed on patients with and without QoL evaluation at 3 months, and a similar pattern of frailty index (fTRST, ASA, G8, Nutritional score, Katz Activities of Daily Living, MiniCog and TUG) was observed among the 2 groups.

Thirty-day mortality occurred in 34 cases (3.7%). Ninety-day and 180-day mortality were 6.3% and 9.0%, respectively. Postoperative morbidity recorded with the CD classification was 39.2% at 30 days and 52.4% at 180 days. Severe complications (CD III-IV) affected 13.5% and 18.7% of all patients at 1 and 6 months after surgery (Supplementary Table 4, available online) Table 4 reports the complication rate by site of primary tumor.

**Table 3.** Univariate and multivariable models for mortality analysis

Baseline covariates	OR for univariate model (95% CI)	*P*	OR for multivariable model (95% CI)	*P*
Age				
≥80 y vs <80 y	1.01 (0.58 to 1.78)	.94	—	—
ASA score^a^				
3-4 vs 1–2	3.27 (1.76 to 6.09)	<.001	—	—
CACI				
≥7 vs 3–6	2.68 (1.54 to 4.63)	<.001	2.46 (1.39 to 4.32)	.002
fTRST^a^				
≥2 vs <2	2.24 (1.30 to 3.87)	.004	—	—
G8 score^a^				
≤14 vs >14	2.80 (1.30 to 6.01)	.008	—	—
PS ECOG^a^				
≥2 vs 0–1	2.57 (1.39 to 4.77)	.003	—	—
Nutritional risk screening				
Mildly impaired vs normal	1.78 (0.94 to 3.35)	.07	1.64 (0.85 to 3.17)	.13
Mod + sev impaired vs normal	3.36 (1.63 to 6.95)	.001	2.67 (1.27 to 5.66)	.01
TUG^a^				
>20 s vs ≤20 s	3.62 (1.51 to 8.67)	.004	—	—
Surgical approach				
Open vs minimally invasive or robotic	2.87 (1.64 to 5.02)	<.001	2.45 (1.35 to 4.46)	.003
Type of surgery^b^				
Palliative vs curative	4.69 (2.19 to 10.00)	<.001	—	—
Site of disease				
Other site of disease vs lower GI	1.42 (0.81 to 2.48)	.21	1.13 (0.61 to 2.08)	.67

^a^ During stepwise process, this variable was not included due to association with CACI. ASA = American Society of Anesthesiologists; CACI = Charlson Age Comorbidity Index; CI = confidence interval; fTRST = Flemish version of the Triage Risk Screening Tool; G8 = Geriatric 8; GI = gastrointestinal; mod + sev = moderately and severely; OR = odds ratio; PS ECOG=Performance Status Eastern Cooperative Oncology Group; TUG = timed up and go.

^b^ During stepwise process, this variable was not included due to association with surgical approach.

**Table 4.** Complication rate sorted by location of the primary tumor^a^

Complications	≤30 d		31–90 d		91–180 d		0–180 d	
	CD I-CD II	CD III-CD IV	CD I-CD II	CD III-CD IV	CD I-CD II	CD III-CD IV	CD I-CD II	CD III-CD IV
	No. (%)	No. (%)	No. (%)	No. (%)	No. (%)	No. (%)	No. (%)	No. (%)
Upper GI (n = 106)	29 (27.4)	18 (17.0)	20 (18.9)	7 (6.6)	15 (14.2)	8 (7.5)	32 (30.2)	23 (21.7)
Lower GI (n = 633)	152 (24.0)	77 (12.1)	94 (14.8)	36 (5.6)	105 (16.6)	30 (4.7)	210 (33.2)	112 (17.6)
HPB (n = 105)	32 (30.5)	21 (20.0)	13 (12.4)	12 (11.4)	14 (13.3)	10 (9.5)	38 (36.2)	26 (24.8)
Thoracic (n = 36)	18 (50.0)	3 (8.3)	12 (33.3)	4 (11.1)	12 (33.3)	2 (5.6)	21 (58.3)	4 (8.3)
Others (n = 68)	11 (16.2)	9 (13.2)	8 (11.8)	6 (8.8)	11 (16.2)	2 (2.9)	15 (22.1)	12 (17.6)

^a^ CD = Clavien-Dindo classification complication grade; GI = gastrointestinal; HBP = pancreas, duodenum, liver, biliary tree.

Age and disease location were not associated with 90-day mortality, but univariate analysis showed that frailty correlated with a higher risk of mortality as detected by the ASA score, CACI, fTRST, G8, ECOG, Nutritional Risk Screening, and TUG. Open surgery was also correlated (Table 3) with higher mortality. On the multivariable analysis, only the CACI of 7 or more (OR = 2.46, 95% CI = 1.39 to 4.32, *P* = .002), moderate or severe malnutrition (OR = 2.67, 95% CI = 1.27 to 5.66, *P* = .01), and open surgery (OR = 2.45, 95% CI = 1.35 to 4.46, *P* = .003) were related to an increased 90-day mortality risk.

## Discussion

The GOSAFE study reports, for the first time to our knowledge, prospective data on how surgery can prolong life while preserving quality in a large cohort of real-world older patients undergoing major cancer surgery. The study also suggests which metrics are likely to be associated with worsening of postoperative QoL. Our analysis highlights how chronological age has a very limited role in predicting outcomes and emphasizes variables that are usually neglected from routine workup strategies.

Focusing research on patient-centered care and evaluating outcomes with a direct impact on patients’ daily lives can result in an efficient and value-based health-care system (29). Published data show that older patients aim for alternative outcomes, that is, activity level, independence, and QoL in addition to oncologic results (30-32). However, designing prospective studies focusing on PROs in the geriatric population presents several challenges: older patients may minimize symptoms, caregivers might be completing PRO scoring sheets on the patient’s behalf, and cognitive impairment and other geriatric syndromes might weaken the accrual and accuracy (33). In the GOSAFE study, the QoL questionnaire EQ-5D was completed in 94.9% of cases, and a specifically designed version was completed by the health-care proxy because of severe cognitive impairment in 50 patients (5.1%). Neither cognitive impairment nor dementia were exclusion criteria from the study, because the validated version of the EQ-5D-3L for cognitively impaired patients was offered. In this study, more than 20% of patients screened positive for cognitive impairment by the MiniCog (≤2), and only 2% of patients reported this using the CACI and 12% by the G8 score. This highlights the potential implications of underappreciating cognitive impairment preoperatively, including the patient’s perioperative decision-making capacity and the risk of postoperative delirium (34).

QoL was chosen as the primary endpoint of this study, but routine frailty screening represents the foundation of personalized care for older cancer patients (35). The GOSAFE provides an accurate insight into a prospectively collected group of 942 patients who were screened for frailty before major cancer surgery. A remarkable finding was the wide variation between the tools: the same patient population proved frail at 5.4% according to the TUG, 49.3% per the ASA, and 68.9% according to G8. A diverse association between the tests we used was noted [ie, QoL and mortality (36,37)]. We also verified that no modification effect or confounding was present between different frailty screening tools. Our results prove that routine frailty screening is feasible: it helps to stratify risks while allowing surgeons to predict postoperative results and counsel patients. Age alone does not correlate with either postoperative mortality or worsening QoL (38). Several frailty screening tools correlate with a higher risk of postoperative mortality at 90 days: CACI of 7 or more, moderate or severe malnutrition, and CD III-IV complications were highly predictive of death. Although specific complications cannot be predicted, surgeons should track and discuss their operative outcomes and use the information about the long-term impact of complications on QoL to guide the communication with patients and families. In addition, frailty screening and nutritional information allow preoperative risk stratification and optimization, help set and understand expectations, and, when appropriate, promote consideration of adapted care.

The GOSAFE study also shows that cancer surgery in older patients is effective in maintaining QoL in addition to benefits of cancer cure and symptoms relief. More than three-quarters of the study population reported equal or improved QoL according to the EQ-5D-3L index. The main domains that statistically significantly improved at 3 to 6 months were anxiety or depression and pain or discomfort, which may otherwise limit independence during the recovery period. Although no standardized increment level was previously validated among geriatric patients undergoing major surgery, other researchers reported an increment of 0.13 in the EQ-5D-3L index as the minimal clinically important difference (from 0.79, SD = 0.20 to 0.82, SD = 0.22 preoperative or at 3 months vs 6 months, *P* < .05) (39).

A limitation of this study is the fact that 135 of 942 patients missed their QoL measurements at 3 and 6 months. The most reported reason was the lack of dedicated study resources to perform tests. This limitation is worth reporting first because it highlights the challenging endeavor of conducting large observational studies with no financial support. In addition, PRO measurement tools used for older patients were not specifically designed for an older surgical population. Although other established instruments such as the EORTC Quality of Life Questionnaire - Elderly Cancer Patients Module (EORTC QLQ-ELD14) (40) would have been valuable alternatives, we prioritized instruments that were quicker to implement and less affected by the limitation of resources. Moreover, we used the EQ-5D-3L, previously validated in large Western population studies, to allow the recruitment of cognitively impaired patients who are frequently encountered in our daily practice. Postoperative mortality was analyzed together with QoL data to appreciate the whole spectrum of outcomes that matter to older patients.

Additionally, logistic regression analysis demonstrated that the type of surgical procedure performed and cancer type does not predict changes in QoL, which could be seen as a limitation due to lack of internal validation. At the same time, the analysis shows that postoperative complications, which vary depending on the type of surgery and cancer site (Table 4), are the key predictors of poor short- and long-term outcomes. To further appreciate these many variables, site-specific subgroup analysis according to cancer type will be performed in future publications. Similarly, a subgroup analysis will be performed comparing palliative vs curative procedures. A third confounding variable is a positive selection bias, where participating centers have a track record of excellence in onco-geriatric management, possibly influencing the outcomes. Similarly, health-care providers involved in the GOSAFE Study might have been “geriatric-minded”, and, despite the limited number of cases where a geriatrician was actively involved (8%), the majority of surgeons were able to offer geriatric-specific management, which is unique (41).

Previously unknown PROs and FR data are highlighted from the GOSAFE. The study shows that cancer surgery in older adults can provide definitive treatment for their cancer while preserving QoL. Anxiety or depression and pain or discomfort are the areas mostly improved by surgical care. Frailty screening tools, history of delirium, palliative surgery, and postoperative complications correlate with a worse QoL at 3 and 6 months after surgery. The predictive tools we have identified should be combined, along with a conversation about the goals of care and measurement of PRO, to direct the preoperative decision on whether to pursue surgery and what kind of surgery to conduct and to help direct patients’ expectations.

## Funding

No funding was received.

## Notes

**Role of the funder:** Not applicable.

**Disclosures:** Authors have no financial disclosure related to the GOSAFE study and its results.

**Author** **contributions:** Conceptualization: GU, IM, AS, MJ, SW, RS, PS, BVL, NLC, NS, FG, AG, RAA. Data curation: FF, CZ, ON, VB. Formal analysis and software: FF, ON. Investigation and Resources: GU, IM, AS, MJ, SW, SR, PS, BVL, NLC, NS, FG, AG and the SIOG surgical task force/ESSO GOSAFE study group. Methodology: GU, IM, AS, MJ, SW, SR, PS, BVL, NLC, NS, FG, AG, FF, CZ. Project administration: CZ, BV. Supervision: IM, GU, SR, BVL, NLC, NS, FG, RAA. Validation and visualization: IM, GU, SR, BVL, NLC, NS, FG, FF, CZ, ON, RAA. Writing—original draft: IM, GU, NS, FG, FF, CZ, RAA. Manuscript review and editing: IM, GU, AS, MJ, SW, SR, PS, BVL, NLC, NS, FG, AG, FF, CZ, ON, RAA.

## Supplementary Material

djac071_Supplementary_Data

## Data Availability

Individual participant data that underlie the results reported in this article, after de-identification, including data dictionaries, will be made available. The data underlying this article will be shared at reasonable request to the principal author. Proposals should be directed to Dr Isacco Montroni (isacmontroni@yahoo.com); information regarding accessing data will be provided upon contact. The study protocol is available in the supplementary material.

## Notes

**Acknowledgements:** Authors would like to thank and acknowledge the pivotal role of the Local Health Authority (AUSL) of Romagna and the Cancer Institute of Romagna (IRST IRCCS), Italy, in collaboration with the European Society of Surgical Oncology (ESSO) and the International Society of Geriatric Oncology (SIOG) Surgical Task Force.

The SIOG surgical task force/ESSO GOSAFE study group: Giovanni Taffurelli, Colorectal surgery Unit, Ospedale “per gli Infermi”, AUSL Romagna, Faenza, Italy; Davide Zattoni, Colorectal surgery Unit, Ospedale “per gli Infermi”, AUSL Romagna, Faenza, Italy; Paola Tramelli, Colorectal surgery Unit, Ospedale “per gli Infermi”, AUSL Romagna, Faenza, Italy; Giacomo Sermonesi, Colorectal surgery Unit, Ospedale “per gli Infermi”, AUSL Romagna, Faenza, Italy; Giorgio Ercolani, Medical and Surgical Sciences Department, Alma Mater Studiorum, University of Bologna, Bologna, Italy; Francesca Tauceri, Department of Oncological Surgery, Morgagni-Pierantoni Hospital, AUSL Romagna, Forlì, Italy; Barbara Perenze, Department of Oncological Surgery, Morgagni-Pierantoni Hospital, AUSL Romagna, Forlì, Italy; Daniela Di Pietrantonio, Department of Oncological Surgery, Morgagni-Pierantoni Hospital, AUSL Romagna, Forlì, Italy; Mariateresa Mirarchi, Department of Oncological Surgery, Morgagni-Pierantoni Hospital, AUSL Romagna, Forlì, Italy; Gianluca Garulli, U.O. Chirurgia Generale, Ospedale “Infermi”, Rimini, AUSL Romagna, Italy; Vincenzo Alagna, U.O. Chirurgia Generale, Ospedale “Infermi”, Rimini, AUSL Romagna, Italy; Andrea Lucchi, Department of Surgery, General surgery Unit, Ceccarini Hospital, AUSL Romagna, Riccione (RN) Italy; Basilio Pirrera, U.O. Chirurgia Generale, Ospedale “Infermi”, Rimini, AUSL Romagna, Italy; Francesco Monari, U.O. Chirurgia Generale, Ospedale “Infermi”, Rimini, AUSL Romagna, Italy; Luigi Conti, Department of Surgery, G.Da Saliceto Hospital, AUSL Piacenza, Piacenza, Italy; Patrizio Capelli, Department of Surgery, G.Da Saliceto Hospital, AUSL Piacenza, Piacenza, Italy; Andrea Romboli, Department of Surgery, G.Da Saliceto Hospital, AUSL Piacenza, Piacenza, Italy; Gerardo Palmieri, Department of Surgery, G.Da Saliceto Hospital, AUSL Piacenza, Piacenza, Italy; Filippo Banchini, Department of Surgery, G.Da Saliceto Hospital, AUSL Piacenza, Piacenza, Italy; Francesca Di Candido, IRCCS Humanitas Research Hospital, Rozzano, Milan, Italy; Michele Carvello, Department of Biomedical Sciences, Humanitas University, Milan, Italy, IRCCS Humanitas Research Hospital, Rozzano, Milan, Italy; Matteo Sacchi, IRCCS Humanitas Research Hospital, Rozzano, Milan, Italy; Francesca De Lucia, IRCCS Humanitas Research Hospital, Rozzano, Milan, Italy; Caterina Foppa, Department of Biomedical Sciences, Humanitas University, Milan, Italy, IRCCS Humanitas Research Hospital, Rozzano, Milan, Italy; Luigi Marano, Department of Medicine, Surgery and Neurosciences, Unit of General Surgery and Surgical Oncology, University of Siena, Italy; Alessandro Spaziani, General, Minimally Invasive and Robotic Surgery, “San Matteo degli Infermi” Hospital, Spoleto, AUSL Umbria2, Italy; Giampaolo Castagnoli, General, Minimally Invasive and Robotic Surgery, “San Matteo degli Infermi” Hospital, Spoleto, AUSL Umbria2, Italy; Alberto Bartoli, General, Minimally Invasive and Robotic Surgery, “San Matteo degli Infermi” Hospital, Spoleto, AUSL Umbria2, Italy; Laura Frain, Division of Thoracic Surgery, Division of Aging, Brigham and Women’s Hospital, Boston, MA, USA; Sam W Fox, Division of Thoracic Surgery, Division of Aging, Brigham and Women’s Hospital, Boston, MA, USA; Kristin Cardin, Division of Thoracic Surgery, Division of Aging, Brigham and Women’s Hospital, Boston, MA, USA; Luis E De Leon, Division of Thoracic Surgery, Division of Aging, Brigham and Women’s Hospital, Boston, MA, USA; Mario Trompetto, Department of Colorectal Surgery, S. Rita Clinic, Vercelli, Italy; Gaetano Gallo, Department of Colorectal Surgery, S. Rita Clinic, Vercelli, Italy; Alberto Realis Luc, Department of Colorectal Surgery, S. Rita Clinic, Vercelli, Italy; Giuseppe Clerico, Department of Colorectal Surgery, S. Rita Clinic, Vercelli, Italy; Giuseppe Sammarco, Department of Medical and Surgical Sciences, University of Catanzaro, Italy; Raffaele De Luca, Department of Surgical Oncology, IRCCS Istituto Tumori “Giovanni Paolo II”, Bari, Italy; Michele Simone, Department of Surgical Oncology, IRCCS Istituto Tumori “Giovanni Paolo II”, Bari, Italy; Rocco Lomonaco, Department of Surgical Oncology, IRCCS Istituto Tumori “Giovanni Paolo II”, Bari, Italy; Michael Fejka, University of Pennsylvania, Perelman School of Medicine, Department of Surgery, Division of Colon and Rectal Surgery, Philadelphia, USA; Joshua IS Bleier, University of Pennsylvania, Perelman School of Medicine, Department of Surgery, Division of Colon and Rectal Surgery, Philadelphia, USA; Matthijs Plas, Department of Surgical Oncology, University Medical Center Groningen, University of Groningen, Groningen, The Netherlands; Hanneke van der Wal-Huisman, Department of Surgical Oncology, University Medical Center Groningen, University of Groningen, Groningen, The Netherlands; Andrea Costanzi, UOC Chirurgia Generale, Ospedale San Leopoldo Mandic, Merate, ASST Lecco, Italy; Giulio Mari, UOC Chirurgia Generale, Ospedale Pio XI, Desio, ASST Brianza, Monza, Italy; Dario Maggioni, UOC Chirurgia Generale, Ospedale Pio XI, Desio, ASST Brianza, Monza, Italy; Roberta Pellegrino, SC Chirurgia Generale—Trapianti Fegato-Pancreas, ARNAS G. Brotzu, Cagliari, Italy; Pietro Ascheri, University Milano Bicocca, Milano, Italy; Jakub Kenig, Department of General, Gastrointestinal, Oncologic Surgery and Transplantology, I Chair of General Surgery Jagiellonian University Medical College, Krakow, Poland; Kinga Szabat, Department of General, Oncologic and Geriatric Surgery, III Chair of General Surgery, Jagiellonian University Medical College, Krakow, Poland; Stefano Scabini, OU General and Oncologic Surgery, Policlinico San Martino, Genova, Italy; Davide Pertile, OU General and Oncologic Surgery, Policlinico San Martino, Genova, Italy; Lorenzo Epis, OU General and Oncologic Surgery, Policlinico San Martino, Genova, Italy; Andrea Massobrio, OU General and Oncologic Surgery, Policlinico San Martino, Genova, Italy; Domenico Soriero, OU General and Oncologic Surgery, Policlinico San Martino, Genova, Italy; Arild Nesbakken, Department of Gatrointestinal Surgery, Oslo University Hospital, Oslo, Norway; Ingeborg Flåten Backe, Department of Gatrointestinal Surgery, Oslo University Hospital, Oslo, Norway; Mariann Lønn, Department of Gatrointestinal Surgery, Oslo University Hospital, Oslo, Norway; Giovanni Ferrari, Unit of Surgical Oncology, ASST Grande Ospedale Metropolitano Niguarda, Milan, Italy; Michele Mazzola, Unit of Surgical Oncology, ASST Grande Ospedale Metropolitano Niguarda, Milan, Italy; Carmelo Magistro, Unit of Surgical Oncology, ASST Grande Ospedale Metropolitano Niguarda, Milan, Italy; Pietro Achilli, Unit of Surgical Oncology, ASST Grande Ospedale Metropolitano Niguarda, Milan, Italy; Alessandro Giani, Unit of Surgical Oncology, ASST Grande Ospedale Metropolitano Niguarda, Milan, Italy; Orestis Ioannidis, 4th Surgical Department, Medical School, Aristotle University of Thessaloniki, Greece; Lydia Loutzidou, 4th Surgical Department, Medical School, Aristotle University of Thessaloniki, Greece; Konstantinos Galanos-Demiris, 4th Surgical Department, Medical School, Aristotle University of Thessaloniki, Greece; Genoveffa Balducci, Sapienza University of Rome, Sant'Andrea University Hospital, Emergency Surgery Unit, Rome, Italy; Barbara Frezza, Sapienza University of Rome, Sant'Andrea University Hospital, Emergency Surgery Unit, Rome, Italy; Alessio Lucarini, Sapienza University of Rome, Sant'Andrea University Hospital, Emergency Surgery Unit, Rome, Italy; Claudia Santos, Hospital Sao Francisco Xavier, Lisbon, Portugal; Filipa Campos Costa, Hospital Sao Francisco Xavier, Lisbon, Portugal; Diogo Cardoso, Hospital Sao Francisco Xavier, Lisbon, Portugal; Isabela Gil, Hospital Sao Francisco Xavier, Lisbon, Portugal; Vasco Cardoso, Hospital Sao Francisco Xavier, Lisbon, Portugal; Lisa Cooper, Rabin Medical Center, Beilinson Hospital, Petach Tikva and Sackler Faculty of Medicine, Tel Aviv University, Tel Aviv, Israel; Baha Siam, Rabin Medical Center, Beilinson Hospital, Petach Tikva and Sackler Faculty of Medicine, Tel Aviv University, Tel Aviv, Israel; Yochai Levy, Rabin Medical Center, Beilinson Hospital, Petach Tikva and Sackler Faculty of Medicine, Tel Aviv University, Tel Aviv, Israel; Baruch Brenner, Rabin Medical Center, Beilinson Hospital, Petach Tikva and Sackler Faculty of Medicine, Tel Aviv University, Tel Aviv, Israel; Hanoch Kashtan, Rabin Medical Center, Beilinson Hospital, Petach Tikva and Sackler Faculty of Medicine, Tel Aviv University, Tel Aviv, Israel; Valerio Belgrano, Department of Surgical Sciences and Integrated Diagnostics (DISC), Policlinico San Martino University General Hospital, University of Genoa, Genova, Italy; Franco Decian, Department of Surgical Sciences and Integrated Diagnostics (DISC), Policlinico San Martino University General Hospital, University of Genoa, Genova, Italy; Beatrice Palermo, Department of Surgical Sciences and Integrated Diagnostics (DISC), Policlinico San Martino University General Hospital, University of Genoa, Genova, Italy; Roberto Eggenhöffner, Department of Surgical Sciences and Integrated Diagnostics (DISC), Policlinico San Martino University General Hospital, University of Genoa, Genova, Italy; Manuela Albertelli, Department of Surgical Sciences and Integrated Diagnostics (DISC), Policlinico San Martino University General Hospital, University of Genoa, Genova, Italy; Luis Sánchez-Guillén, Colorectal and Gastrointestinal Department, Hospital General Universitario de Elche, Universidad Miguel Hernández, Alicante, Spain; Antonio Arroyo, Colorectal and Gastrointestinal Department, Hospital General Universitario de Elche, Universidad Miguel Hernández, Alicante, Spain; Francisco López-Rodríguez, Colorectal and Gastrointestinal Department, Hospital General Universitario de Elche, Universidad Miguel Hernández, Alicante, Spain; Sandra Lario, Colorectal and Gastrointestinal Department, Hospital General Universitario de Elche, Universidad Miguel Hernández, Alicante, Spain; Cristina Lillo, Colorectal and Gastrointestinal Department, Hospital General Universitario de Elche, Universidad Miguel Hernández, Alicante, Spain; Minas Baltatzis, HPB Unit, Manchester Royal Infirmary, University of Manchester, UK; Anthony KC Chan, HPB Unit, Manchester Royal Infirmary, University of Manchester, UK; Ajith K Siriwardena, HPB Unit, Manchester Royal Infirmary, University of Manchester, UK; Giovanna Da Silva, Department of Colorectal Surgery, Cleveland Clinic, Florida, US.

**Prior presentations:** GOSAFE Interim results were previously podium presented during the ASCO meeting in 2019 and virtually in 2020.
