# Correction to: Quality of Life in Older Adults After Major Cancer Surgery: The GOSAFE International Study

**DOI:** 10.1093/jnci/djae092

**Published:** 2024-04-29

This is a correction to: Isacco Montroni, Giampaolo Ugolini, Nicole M Saur, Siri Rostoft, Antonino Spinelli, Barbara L Van Leeuwen, Nicola De Liguori Carino, Federico Ghignone, Michael T Jaklitsch, Ponnandai Somasundar, Anna Garutti, Chiara Zingaretti, Flavia Foca, Bernadette Vertogen, Oriana Nanni, Steven D Wexner, Riccardo A Audisio, the SIOG Surgical Task Force/ESSO GOSAFE Study Group, Quality of Life in Older Adults After Major Cancer Surgery: The GOSAFE International Study, *JNCI: Journal of the National Cancer Institute*, Volume 114, Issue 7, July 2022, Pages 969–978, https://doi.org/10.1093/jnci/djac071

In the online version of this manuscript, the file within the **Supplementary data** section needs to be replaced due to the change, at page 5, of the EQ-5D-3L specimen. The correct file should read as in the linked file here replacing djac071_Supplementary_Data—pdf file in the original publication:

https://academic.oup.com/jnci/article/114/7/969/6565327#supplementary-data

The emendation is made only in this correction notice to preserve the version of record.

## Supplementary Material

djae092_Supplementary_Data

