# Supplementary material for: Quality of Life in Older Adults After Major Cancer Surgery: The GOSAFE International Study
Source: J Natl Cancer Inst. 2022 Apr 8;114(7):969–78. doi: 10.1093/jnci/djac071 (PMC9275771; doi:10.1093/jnci/djac071)
Supplement: djac071_Supplementary_Data [file djac071_supplementary_data.pdf]

Supplementary Table 1. Recruiting international centers

Institution	Department	City	Country	Activation date
Ospedale “per gli Infermi” AUSL Romagna (Coordinating Center)	U.O. Chirurgia Generale	Faenza (RA)	Italy	15-Feb-17
Ospedale “GB. Morgagni-L. Pierantoni” AUSL Romagna	U.O. Chirurgia Generale e Terapie Oncologiche Avanzate	Forlì (FC)	Italy	15-Feb-17
Ospedale “Ceccarini”, AUSL Romagna	U.O. Chirurgia Generale	Riccione (RN)	Italy	09-May-17
AUSLPiacenza, PO Piacenza	U.O. Chirurgia generale	Piacenza	Italy	11-July-17
Humanitas Clinical and Research Center	Division of Colon and Rectal Surgery,	Rozzano (MI)	Italy	14-July-17
Ospedale “S. Matteo degli Infermi” AUSL Umbria-2	General, Minimally Invasive and Robotic Surgery	Spoleto (PG)	Italy	26-July-17
Brigham and Women's Hospital	Thoracic Surgery	Boston (MA)	USA	11-Oct-17
Clinica S. Rita	Department of Colorectal Surgery	Vercelli	Italy	18-Oct-17
Istituto Tumori Giovanni Paolo II IRCCS	Department of Surgical Oncology	Bari	Italy	22-Nov-17
University of Pennsylvania, Perelman School of Medicine	Department of Surgery	Philadelphia (PA)	USA	22-Nov-17
University Medical Center Groningen	Department of Surgical Oncology	Groningen	Netherlands	30-Nov-17
ASST Grande Ospedale Metropolitano Niguarda	Chirurgia generale Oncologia e Mininvasiva	Milan	Italy	29-Nov-17
Jagiellonian University Medical College	Department of General, Oncologic and Geriatric Surgery	Krakow	Poland	12-Dec-17
Ospedale Policlinico S. Martino IRCCS	OU General and Oncologic Surgery	Genova	Italy	06-Dec-17
Oslo University Hospital	Department of Surgery	Oslo	Norway	12-Dec-17
ASST Monza - Ospedale di Desio	General and Emergency Surgery	Desio (MB)	Italy	18-Dec-17
Aristotle University of Thessaloniki, Medical School	4th Surgical Department	Thessaloniki	Greece	23-Jan-18
Hospital Universitario y Politécnico La Fe	General and Digestive surgery	Valencia	Spain	09-Feb-18
Roger William Medical Centre Providence	Surgical Oncology	Providence (RI)	USA	27-Feb-18
Sapienza University of Rome, Sant'Andrea University Hospital	Emergency Surgery Unit	Rome	Italy	27-Mar-18
Hospital Sao Francisco Xavier	General Surgery	Lisbon	Portugal	04-Apr-18
Rabin Medical Center	Department of Geriatrics	Tel Aviv	Israel	26-Apr-18
Ospedale Policlinico S. Martino IRCCS	Department of Surgical Sciences and Integrated Diagnostics (DISC)	Genova	Italy	14-May-18
Hospital General Universitario de Elche, Universidad Miguel Hernández	Colorectal & Gastrointestinal Department	Alicante	Spain	21-May-18
Manchester Royal Infirmary, University of Manchester	HPB Unit	Manchester	UK	16-July-18
Cleveland Clinic Foundation	Department of Colorectal Surgery	Weston (FL)	USA	11-Jan-19

Supplementary Table 2. Functional assessment indicators

Test	Acronym	Range of possible scores	Frailty indicator threshold	Purpose
EQ 5D-3L	EQ 5D-3L Index	0-1	Not applicable	Evaluation of QoL assessing patient's mobility, self-care, usual activities, pain and anxiety, includes a visual scale
EQ 5D-3L	EQ 5D-3L VAS	0-100	Not applicable	
Eastern Collaborative Oncology Group Performance Status	ECOG PS	0-4	≥1	Evaluation of cancer burden on functional status
Katz Activities of Daily Living	ADL	0-6	<5	Evaluation of functional independence
Mini-Cog	Mini-Cog	0-5	≤2	Detection of cognitive impairment in older adults therefore suitable for a more thorough evaluation.
Flemish version of the Triage Risk Screening Test	fTRST	0-6	≥2	Detection of hospitalized geriatric patients at risk for frailty
Timed Up & Go Test	TUG	Not applicable	≥20 sec	Three-meters walking test to evaluate functional status
Geriatric 8	G8	0-17	≤14	Detection of onco-geriatric patients who may benefit from comprehensive geriatric assessment
Nutritional Risk Screening	NRS	Normal to severely impaired nutritional status	Moderately to severely impaired	Evaluation of nutritional status taking into account BMI, weight loss and food intake
American Society of Anesthesiology score	ASA	1-5	Not applicable	Evaluation of preoperative general clinical condition and estimation of anesthesiologic risk
Charlson Age Comorbidity Index	CACI	0-42	≥6	Evaluation of cumulative burden of patient's comorbidities

Supplementary Table 3. Type of surgical procedures performed

Procedure	Frequency
Colectomy (left-right-subtotal)	434
Low anterior resection of the rectum	144
Abdominoperineal resection	44
Small bowel resection	5
Adrenalectomy	4
Nephrectomy	5
Cystectomy	3
Ureter resection	1
Prostatectomy	4
Hepatectomy (segmental-lobectomy)	48
Hepatectomy (wedge)	11
Common bile duct resection	3
Lung lobectomy	29
Lung wedge resection	3
Chest wall resection	2
Gastrectomy total	25
Gastrectomy subtotal	67
Esophagectomy	12
Whipple	35
Pancreatectomy distal	4
Splenectomy	2
Sarcoma excision	5
Other	52
Total	942

Supplementary Table 4. Postoperative complications

Complications	≤30 days		31-90 days		91-180 days		0-180 days	
	CD*I-CDII n (%)	CDIII-CDIV n (%)	CDI-CDII n (%)	CDIII-CDIV n (%)	CDI-CDII n (%)	CDIII-CDIV n (%)	CDI-CDII n (%)	CDIII-CDIV n (%)
Patients with at least one complication (CD III-IV)	/	128 (13.5)	/	65 (6.9)	/	52 (5.5)	/	176 (18.7)
Patients with at least one complication (CD I-IV)	370 (39.2)		212 (22.5)		210 (22.2)		494 (52.4)	
Respiratory	53 (5.6)	34 (3.6)	24 (2.6)	13 (1.4)	26 (2.9)	15 (1.7)	69 (7.7)	45 (5.0)
Cardiac	35 (3.7)	20 (2.1)	14 (1.5)	13 (1.4)	16 (1.8)	8 (0.9)	44 (4.9)	37 (4.1)
Renal	54 (5.7)	7 (2.1)	15 (1.5)	3 (1.4)	10 (1.8)	2 (0.9)	66 (4.9)	12 (4.1)
Neurological	25 (2.7)	1 (0.1)	8 (0.9)	6 (0.7)	9 (1.0)	2 (0.2)	38 (4.2)	8 (0.9)
Nutritional	24 (2.5)	4 (0.4)	11 (1.2)	1 (0.1)	10 (1.1)	3 (0.3)	36 (4.0)	7 (0.8)
Pressure sores	2 (0.2)	2 (0.2)	1 (1.0)	0 (0.0)	0 (0.0)	1 (0.1)	2 (0.2)	3 (0.3)
Pain	6 (0.6)	2 (0.2)	1 (0.1)	2 (0.2)	4 (0.4)	2 (0.2)	10 (1.1)	3 (0.3)
Delirium	11 (1.2)	0 (0.0)	3 (0.3)	0 (0.0)	0 (0.0)	0 (0.0)	13 (1.5)	0 (0.0)
Wound	44 (4.7)	14 (1.5)	29 (3.2)	6 (0.7)	32 (3.6)	1 (0.1)	82 (9.2)	16 (1.8)
Gastrointestinal	65 (6.9)	52 (5.5)	46 (5.0)	19 (2.1)	38 (4.2)	17 (1.9)	110 (12.3)	73 (8.1)
Other complication	73 (7.7)	31 (3.3)	41 (4.5)	15 (1.6)	39 (4.4)	11 (1.2)	153 (17.1)	57 (6.4)

*CD: Clavien-Dindo classification complication grade

Supplementary Figure 1. The EQ-5D-3L.

**MOBILITY**	I have no problems in walking about	☐
	I have some problems in walking about	☐
	I am confined to bed	☐
**SELF-CARE**	I have no problems with self care	☐
	I have some problems washing or dressing myself	☐
	I am unable to wash or dress myself	☐
**USUAL ACTIVITIES**	I have no problems with performing my usual activities	☐
	I have some problems with performing my usual activities	☐
	I am unable to do my usual activities	☐
**PAIN/ DISCOMFORT**	I have no pain or discomfort	☐
	I have moderate pain or discomfort	☐
	I have extreme pain or discomfort	☐
**ANXIETY/ DEPRESSION**	I am not anxious or depressed	☐
	I am moderately anxious or depressed	☐
	I am extremely anxious or depressed	☐

YOUR HEALTH TODAY

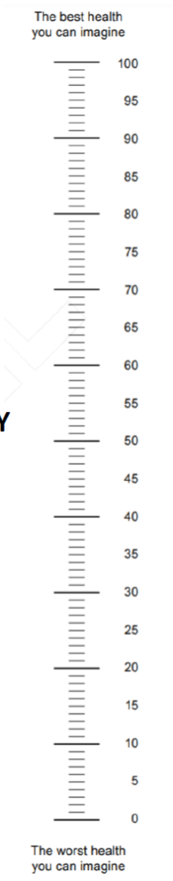

Supplementary Methods: study protocol

esso
THE EUROPEAN SOCIETY OF SURGICAL ONCOLOGY

SIOG
INTERNATIONAL SOCIETY OF GERIATRIC ONCOLOGY

GO SAFE Study

Geriatric Oncology Surgical Assessment and Functional rEcovery after Surgery

An international prospective audit to evaluate postoperative functional outcomes and quality of life after cancer surgery in geriatric patients

Date and Version: 15/03/2017 - Amendment 1.0

GO SAFE

KEY TRIAL CONTACTS

**Chief Investigator:**	**Prof. Giampaolo Ugolini**<br>Alma Mater University of Bologna<br>Ospedale degli Infermi, Faenza (RA), IT<br>General Surgery Unit<br>Telephone +39 349 4347576<br>e-mail address: g.ugolini@unibo.it
**Co-Investigators:**	**Dr. Barbara Van Leeuwen**<br>Dept. of Surgical Oncology<br>UMCG, Groningen, NL
**Sponsor/Promoter:**	**AUSL della Romagna**<br>Via de Gasperi 8<br>48121 Ravenna
**Study management:**	**Unità di Biostatistica e Sperimentazioni Cliniche**<br>Istituto Scientifico Romagnolo per lo Studio e la Cura dei Tumori (IRST) s.r.l IRCCS<br>Via Piero Maroncelli, 40/42<br>47014 Meldola (FC)<br>Telephone + 390544285813 Fax +390544285330<br>e-mail address: cc.ubsc@irst.emr.it
**Statistician:**	**Dr. Oriana Nanni**<br>Istituto Scientifico Romagnolo per lo Studio e la Cura dei Tumori (IRST) s.r.l IRCCS<br>Via Piero Maroncelli, 40/42<br>47014 Meldola (FC)<br>e-mail address: oriana.nanni@irst.emr.it

Steering committee:

Riccardo Audisio
Dept. of Surgical Oncology - St Helens Hospital Liverpool, UK

Nicola De Liguori Carino
HBP Surgery, Manchester Royal Infirmary, UK

Giorgio Ercolani
Alma Mater University of Bologna - General Surgery Unit - Ospedale Morgagni, Forlì, IT

Nadav Haim
Colorectal surgery Sheba Medical Center Ramat Gan, HaMerkaz, Israel

Michael Jaklitsch
Dept of Thoracic Surgery - Brigham and Women's Hospital, Boston, MA, USA

Isacco Montroni
General Surgery Unit - Ospedale degli Infermi, Faenza, IT

Siri Rostoft
Dept. of Geriatric Oncology, Oslo University Hospital, NW

Ponnandai Somasundar
Dept. of Surgical Oncology, Roger Williams Medical Center Providence - Boston University, RI, USA

Promoted by SIOG surgical task force and ESSO

Confidentiality Statement

This document contains confidential information that must not be disclosed to anyone other than the Sponsor/Promoter, the Investigator’s Team, IRST IRCCS, regulatory authorities, and members of the Ethics Committee.

Protocol approval and Investigator agreement

Geriatric Oncology Surgical Assessment and Functional rEcovery after Surgery

The undersigned agree and confirm that:

The following protocol has been agreed and accepted and the Chief Investigator agrees to conduct the trial in compliance with the approved protocol and will adhere to the principles outlined in ICH GCP guidelines, Sponsor/Promoter SOP's and other regulatory requirements as amended.

The confidential information contained in this document will not be used for any other purpose other than the evaluation or conduct of the clinical investigation without the prior written consent of the Sponsor/Promoter.

The findings of the study will be made publically available through publication or other dissemination tools without any unnecessary delay and that an honest accurate and transparent account of the study will be given; and any discrepancies from the study as planned in this protocol will be explained.

Giampaolo Ugolini	______________________	____________
Chief Investigator	Signature	Date
Oriana Nanni	______________________	____________
Trial Statistician	Signature	Date

By signing this document I am confirming that I have read the protocol for the above study and I agree to conduct the study in compliance with the protocol and ICH GCP.

________________	______________________	____________
Principal Investigator	Signature	Date

ABBREVIATIONS

AE	Adverse event
AR	Adverse reaction
CC	Coordinating Center
CI	Chief Investigator
CRA	Clinical Research Associate (Monitor)
CRF	Case Report Form
CRO	Contract Research Organisation
CT	Clinical Trials
CTC	Common toxicity criteria
ECOG	Performance status (Eastern Cooperative Oncology Group, ECOG Scale)
FR	Functional recovery
GCP	Good Clinical Practice
IB	Investigators Brochure
ICF	Informed Consent Form
ICH	International Conference of Harmonisation
IDMC	Indipendent Data Monitoring Committee
IEC	Independent Ethics Committee
IMP	Investigational Medicinal Products
IRB	Independent Review Board
PI	Principal Investigator
QoL	Quality of Life
RECIST	Response Evaluation Criteria In Solid Tumors
SAE	Serious Adverse Event
SAR	Serious Adverse Reaction
SOP	Standard Operating Procedure
SUSAR	Suspected Unexpected Serious Adverse Reactions
WHO	World Health Organization

TABLE OF CONTENTS

KEY TRIAL CONTACTS **2**

STEERING COMMITTEE: **3**

1. BACKGROUND **8**

2. RATIONALE **9**

3. AIM OF THE STUDY **9**

3.1 Primary Objective 10

3.2 Secondary Objectives 10

4. STUDY PROTOCOL **10**

4.1 Centres and Investigators 10

4.2 Study Design 11

4.3 Study Population 11

4.3.1 Inclusion Criteria 11

4.3.2 Exclusion criteria 11

4.4 Local approvals 12

5. PROCEDURES AND DATA COLLECTION **12**

5.1 Informed Consent 12

5.2 Registration (CRF A) 12

5.3 Data Collection 13

5.3.1 Baseline evaluation (CRF B) 13

5.3.2 Operative details and early postoperative outcome (CRF C) 14

5.3.3 Follow up (CRF 3M-6M) 15

5.4 Study plan flowsheet and CRF completion times 16

5.5 Confidentiality 16

5.6 Data quality assurance 17

6 STATISTICAL CONSIDERATIONS **17**

6.1 Data analysis 17

6.2 Sample size 17

6.3 Study duration 18

7 WITHDRAWAL OF PATIENTS FROM THE STUDY **18**

8 ETHICAL ASPECTS 18

8.1 LOCAL REGULATIONS/DECLARATION OF HELSINKI 18
8.2 INDEPENDENT ETHICAL COMMITTEE 18
8.3 INFORMED CONSENT 19
8.4 PATIENT DATA PROTECTION 19

9 ADMINISTRATIVE REGULATIONS 19

9.1 CURRICULUM VITAE 20
9.2 SECRECY AGREEMENT 20
9.3 FINANCIAL ARRANGEMENTS 20

10 OWNERSHIP OF THE DATA AND USE OF THE STUDY RESULTS.........................20

11 PUBLICATION POLICY AND AUTHORSHIP...20

12 PROTOCOL AMENDMENTS 21

13 REFERENCES 22

1. BACKGROUND

Progressive aging of the world population has become one of the most significant challenges for national health care systems. With aging, the incidence and prevalence of cancer increases: it has been estimated that in 2020 more than 60% of all malignancies will occur in patients aged 70-year and older. At the same time, progress in medical knowledge has determined an extremely positive impact in clinical practice. In particular, improvements in perioperative care, surgical minimally invasive techniques and the introduction of multimodal treatment have made surgery feasible for a higher number of patients. Nevertheless, several studies show that senior adults affected by cancer are often treated sub-optimally, above all in the surgical field.

It is well known that onco-geriatric patients are at higher risk of developing postoperative complications because they are often affected by multiple comorbidities. It has been reported that up to 80% of elderly patients might experience a surgical complication. Thus, after major surgery, patients may be at risk of both developing postoperative complications, and suffering major discomfort that can negatively affect postoperative quality of life.

The vast majority of research studies are focused on short-term outcomes and do not explore long- term disability or postoperative quality of life.

Onco-geriatric patients represent a challenge for surgical oncologists because, despite the evidence that comorbidities are often responsible for poor postoperative outcomes, patients' selection has not been completely standardized yet. Preoperative assessment of the functional status is fundamental to identify fit, vulnerable and frail individuals in order to avoid under- or over-treatment. Functional recovery has been shown to be of critical value in the elderly population since restoration/conservation of independence is probably the most important end-point for senior adults. Individualization of elderly cancer-patients care is closely related with the possibility of preserving their functional capacity. We could conclude that for elderly patients, perhaps more than anyone else, "quality" is more important than "quantity" of life.

2. RATIONALE

We aim to improve outcomes of onco-geriatric patients' surgical management. Our research project will focus on quality of life and functional recovery after surgery. The most important expected result will be the collection of data that clinicians will be able to exploit in the management of frail and 'pre-frail' patients with the potential to reduce disparities in elderly patient care. In addition, the 'multidisciplinary work ethic' in the management of this specific group of patients, regardless for their primary condition, will be extensively promoted to determine small but clinically important incremental improvements in elderly care.

We need to conduct the GO SAFE study for several reasons:

- There is a dramatic lack of knowledge on elderly cancer surgical patients
- Although survival is commonly reported after surgery, quality of life (QoL) and functional recovery (FR), including nutritional status, are rarely measured
- To promote the practice of a multidisciplinary management of elderly cancer patients
- To understand how frailty, comorbidities and malnourishment are associated with early and long-term clinical outcomes after surgery in elderly cancer patients
- To obtain prospective data to assist clinicians in tailoring the care, avoiding under/over-treatment
- To identify new strategies to improve functional outcomes (as cardiorespiratory/nutritional prehabilitation)

To identify areas warranting further research studies and surgical audit in the older adults cancer population

3. AIM OF THE STUDY

GO SAFE study is a prospective international collaborative high-quality registry aiming to gain knowledge about postoperative outcomes in older cancer patients with a particular emphasis on QoL and FR. The target is to obtain meaningful data to assist clinicians in tailoring the care,

avoiding under/over-treatment, providing robust data to identify new strategies to improve functional outcomes in older cancer patients.

3.1 Primary Objective

To evaluate the effects of surgery on patients' life perception by comparing pre- and post-operative QoL in elderly patients undergoing major surgery for solid malignancies using a self-reported Quality of Life assessment tool (EQ 5D-3L)

3.2 Secondary Objectives

- To evaluate FR in terms of nutritional status, restoration of daily activities (ADL) and cognitive status (Mini-Cog)
- To evaluate 3 and 6 months postoperative morbidity and mortality
- To obtain prognostic factors for postoperative functional recovery which will assist in the treatment planning /intervention of future elderly patients who are offered surgery for cancer
- To identify variables affecting postoperative quality of life

4. STUDY PROTOCOL

4.1 Centres and Investigators

We aim to involve in the study as many centers as possible. All surgical units performing cancer surgery in elderly patients are invited to participate. Participating investigators will be surgical oncologists. Each center will require approval from local Institutional Review Board and/or the Ethic Committee before starting to enroll patients.

Investigators will be responsible to obtain a written informed consent from each eligible patient in according to the local IRB regulation, ahead of surgery (please see note below in case of demented patients). Every center shall commit to send clinical data after the 6-month follow up.

Periodic evaluation of patient data entry, centers' activity, and cohesion of the centers will be shared among the investigators.

4.2 Study Design

GO SAFE is a multicenter international observational prospective cohort study. The study is non-for-profit. Recruiting centers will collect data prospectively. Recruited patients will be followed for 6 months after their surgery. The original treatment plan, as designed by each individual recruiting centre, will not be altered or affected by the study inclusion.

4.3 Study Population

Centers should ensure that they would make every possible effort to include all consecutive eligible patients during the study period and provide completeness of data entry to ensure a 'real-life' study.

4.3.1 Inclusion Criteria

1. All consecutive patients, both gender, aged ≥70
2. Patients affected by solid malignancy
3. Patients undergoing elective major surgical procedures with curative or palliative intent (all major procedures including any resection, for any cancer, via any operative approach, open, laparoscopic, robotic, etc…)
4. Informed consent obtainment

4.3.2 Exclusion criteria

1. Patients undergoing emergent/urgent surgical procedures
2. Planned hospital stay less than 48 hours

4.4 Local approvals

Inclusion in the study does not imply any deviation from the current standard of practice, and no change is expected to the perioperative treatment at any point. Patients will be only asked to complete simple screening/assessment tests: for this reason this study should be registered as a prospective observational study at each participating hospital IRB. It is the responsibility of the local team to ensure that regulatory process is completed for its hospital. Participating centres will be asked to confirm that they have gained formal approval and to provide an Identification Number.

5. PROCEDURES AND DATA COLLECTION

5.1 Informed Consent

It is the responsibility of the investigator, or a person designated by the investigator, to obtain (if applicable) written informed consent from each individual participating in this study. When applicable, each patient/health care proxy must sign and date the latest approved version of the Informed Consent form before any study specific procedures are performed.

Patients must receive an explanation that they are completely free to refuse to enter in this study and to withdraw from it at any time and for any reason without prejudice to future care, and with no obligation to give the reason for withdrawal. The original signed form will be retained at the study site. A copy of the informed consent form will be delivered to the patient. A form for obtaining written informed consent for this observational study will be provided.

5.2 Registration (CRF A)

All patients for whom eligibility criteria have been verified will be registered by each participating center in the eCRF.

The following data will be collected at registration:

- Patient’s Date of birth
- Patient’s Gender
- Date of Informed consent
- Date of registration

- Center informations

5.3 Data Collection

Clinical reporting forms (CRFs) have been designed to be completed along the normal daily-life practice, trying to minimize the 'extra work' for local investigators. Tests, carried out at base-line and follow-up evaluation, could be easily completed by surgical trainees, medical students and nurses adequately trained. Surgical data analysis, including detection of postoperative complications, should require the supervision of an attending/consultant surgeon.

CRFs are to be completed through use of an EDC system. Sites will have access to a manual for appropriate CRF completion. All CRFs should be completed by designated, trained site staff. CRFs should be reviewed and electronically signed and dated by the investigator or a designee.

If a correction is required for an CRF, the EDC system will create an electronic audit trail.

Participants must maintain quality of their database and update their database. Access to the raw data will be according to agreement of both parties. Each center must keep a file of all consecutive patients that have been entered in the database for random quality monitoring.

5.3.1 Baseline evaluation (CRF B)

For every eligible patient, demographic data will be collected followed by a fast preoperative functional assessment including:

- Charlson Comorbidity Index
- "Timed Up and Go" test
- Nutritional Risk Screening (NRS)
- American Society of Anesthesiology (ASA) score
- ECOG Performance Status
- G8 geriatric screening tool
- Mini-Cog
- Activities of Daily Living (ADL)
- Quality of Life (EQ 5D-3L, Self or Proxy-1 version).

- History of delirium during illness or hospital admission
- History of Smoking
- History of falls in the 6 months prior to the operation
- Living situation
- Lab's (Albumin, Hemoglobin, Creatinin)
- Polipharmacotherapy (total number of medications)
- Preoperative chemotherapy/radiation therapy
- Involvement of geriatric specialist in preoperative care

5.3.2 Operative details and early postoperative outcome (CRF C)

Data regarding surgical procedures and perioperative measures will be collected. Complications will be reported and graded according to Clavien-Dindo Classification.

- Cancer site
- Surgical Procedure Category: Ortho, Gyn, Breast, Upper-GI, Colorectal, HBP, Peritoneum, Thoracic (esophagus), Head & Neck, Urology
- Type of procedure (describe)
- Type of anesthesia (General, Spinal, Epidural)
- Type of surgery (palliative, curative)
- Duration of anaesthesia (min)
- Surgical approach (Open/Laparoscopic/Robotic….)
- Need of ICU stay (Y/N; n. days)
- Perioperative blood transfusions (Units of Packed RBC's) within the surgical admission
- Postoperative length of stay (days in surgical unit)
- Patient discharged to same preoperative setting
- Patient transferred to Medicine/Rehabilitation facility
- Tumor stage (TNM/Stage)

- Involvement of geriatric specialist in postoperative care
- 30 day morbidity (Clavien-Dindo)
- 30 day mortality

5.3.3 Follow up (CRF 3M-6M)

Three- and six-month follow up data will be collected after surgery within a range of 2 weeks from the due date.

Data	3 months	6 months
• Morbidity (Clavien-Dindo)	X	X
• Mortality	X	X
• Living situation	X	X
• Weight	X	X
• Nutritional Screenig	X	X
• "Timed Up and Go" test	X	X
• Mini-Cog	X	X
• ECOG Performance Status	X	X
• ADL	X	X
• Self-reported Quality of Life (EQ 5D-3L)	X	X
• Postoperative Chemotherapy	X	X
• Postoperative Radiation Therapy	X	X
• Rehabilitation program	X	X
• Nutritional supplement	X	X

• Involvement of a geriatric specialist	X	X

Local investigators should be also proactive in identifying postoperative events. For example they may review patients notes during admission and before discharge, as well as they could review hospital and outpatient clinic systems to check for readmission and/or other unplanned events.

5.4 Study plan flowsheet and CRF completion times

REGISTRATION	CRF A
PREOPERATIVE ASSESSMENT (BASELINE EVALUATION) ↓	CRF B
OPERATIVE DETAILS EARLY POSTOPERATIVE OUTCOME (1 month) ↓	CRF C
FOLLOW UP 3 months	CRF 3M
6 months	CRF 6M

5.5 Confidentiality

Personal patients' data will not be shared to anyone outside of the research team. The information collected in this research project will be kept private. All patient information will be anonymized. The database used is certified, highly secured and is stored in a encrypted server that meets all the requirements for data-safety and privacy set by international law.

5.6 Data quality assurance

- Medical review with investigators
- CRF quality check, query firing, data cleaning
- Early feedback with local research team via teleconferences

6 STATISTICAL CONSIDERATIONS

6.1 Data analysis

The Full Analysis Set (FAS) consists of all registered patients.

The primary endpoint will be measured for all registered subjects who fulfill preoperative and postoperative EQ VAS. Demographic and baseline patient characteristics will be summarized for all patients in the FAS. Continuous-scaled variables (e.g., age) will be summarized with means, medians, standard deviations, quartiles, and minimum and maximum values. Categorical variables (e.g., sex) will be summarized using patient counts and percentages. Study endpoints and variables will be evaluated using descriptive statistics, and the key figures of the distributions will be presented in tables. Univariate analyses will allow for a first overview of potentially influential factors.

Multiple linear regression models will be performed in order to evaluate predictors of functional recovery at 3 months and 6 months after surgery.

Exploratory subgroup analyses will be performed. Missing values will be replaced and estimated using multiple imputations. Furthermore, sensitivity analysis will be executed using complete-case analysis.

6.2 Sample size

A sample size of 265 patients who completed pre and postoperative EQ VAS questionnaires will have a 90% power to detect an effect size of 0,2 between pre and post surgery ,using a paired t-test with a 0,05 two sided significance level.

Given a potential loss to follow-up (about 10%), uncompleted questionnaires (about 10%) and postoperative mortality (about 15%), the sample size will be increased to 350-400 patients (see

ref 18 and 19).

6.3 Study duration

Enrollment period: 24 months

Follow- up: 6 months

Data analysis : 6 months

Total duration of the study: 36 months

7 WITHDRAWAL OF PATIENTS FROM THE STUDY

Patients have the right to withdraw at any time for any reason during their participation in this observational study.

8 ETHICAL ASPECTS

8.1 Local regulations/Declaration of Helsinki

The responsible Investigator will ensure that this study is conducted in compliance with the protocol, following the instructions and procedures described, adhering to the principles of Good Clinical Practice ICH Tripartite Guideline (December 2000) and in accordance with the principles laid down by the 18th World Medical Assembly (Helsinki, 1964 and further amendments) or with the laws and regulations of the country in which the research is conducted, whichever affords the greater protection to the individual.

8.2 Independent Ethical Committee

The protocol, informed consent and any accompanying material provided to the patient will be submitted by the investigator to an Independent Ethical Committee for review. Approval from the committee must be obtained before starting the study. Any modifications made to the protocol, informed consent or material provided to the patient after receipt of the Ethics Committee approval must also be submitted by the investigator to the Committee in accordance with local procedures and regulatory requirements. The Independent Ethical

Committee approval report must contain details of the trial (title, protocol number and version), documents evaluated (protocol, informed consent, accompanying material) and the date of the approval.

8.3 Informed Consent

It is the responsibility of the Investigator to obtain written informed consent from each subject prior to entering the trial or, where relevant, prior to evaluating the subject's suitability for the study.

The informed consent document used by the Investigator for obtaining the subject's informed consent must be reviewed and approved by the Ethical Committee.

A copy of the patient's signed written consent will be kept by the center in the proper section of the Investigator Site File.

8.4 Patient data protection

The Informed Consent Form will incorporate wording that complies with relevant data protection and privacy legislation. In agreement with this wording, patients will authorize the collection, use and disclosure of their study data and samples by the Investigator and by those persons who need that information for the purposes of the study.

The Informed Consent Form will explain that the study data will be stored in a computer data base, maintaining confidentiality in accordance with national data legislation.

The Informed Consent Form will explain that the samples obtained by patients will be anonymized and stored in accordance with national data legislation.

The Informed Consent Form will also explain that for data verification purposes, authorized representatives of Sponsor/Promoter, a regulatory authority, an Ethics Committee may require direct access to parts of the hospital or practice records relevant to the study, including patients' medical history.

9 ADMINISTRATIVE REGULATIONS

The Coordinating Center (CC) is responsible for drawing up the final version of the protocol, implementing the CRFs and the electronic database, defining general

organizational procedures and organizing periodic meetings and newsletters. The CC will also undertake the following: support for the preparation of all documents needed for EC submission of the study protocol for each participating center, training of staff assigned to data collection, definition of monitoring procedures.

9.1 Curriculum vitae

An updated copy of the curriculum vitae of each Principal Investigator, duly signed and dated, will be provided to the CC prior to the beginning of the study.

9.2 Secrecy agreement

All goods, materials, information (oral or written) and unpublished documentation provided to the Investigators, including this protocol and the case report forms, shall be considered confidential and may not given or disclosed to third parties.

9.3 Financial Arrangements

This is a non-for-profit study promoted by SIOG surgical task force and ESSO. No registration fee is requested to participate to the GO SAFE study.

No financial reimbursements will be made to participating centers/investigators.

10 OWNERSHIP OF THE DATA AND USE OF THE STUDY RESULTS

Participants shall retain the ownership of their own data. Each participating center is responsible for accurate data entry and has access to their data only. No data sharing will be performed with any third party. Personal data will be anonymized and confidential encrypted in a secure place. Professional support for data analysis will be made available.

11 PUBLICATION POLICY AND AUTHORSHIP

Clinical results will be published collaboratively. Interim and final analysis will be presented at scientific conferences to ensure visibility.

Data will be published, acknowledging authorship to all the centers giving a substantial contribution, under the name of ”SIOG (International Society of Geriatric Oncology)

surgical task force/ESSO (European Society of Surgical Oncology) GO SAFE study group”.

A maximum of 5 investigators from each individual surgical unit will be included as formal co-investigators in this research, and will be PubMed searchable and citable. The output from this research will be published on behalf of the ”SIOG (International Society of Geriatric Oncology) surgical task force/ESSO (European Society of Surgical Oncology) GO SAFE study group”.

Each hospital may participate with different surgical units (GI, HBP, etc…) and each unit should enrol a minimum number of 20 patients in order to claim authorship.

12 PROTOCOL AMENDMENTS

It is specified that the appendices, attached to this protocol and referred to in the main text of this protocol, form an integral part of the protocol.

No changes or amendments to this protocol may be made by the Investigators after the protocol has been agreed to and signed by both parties . Any change agreed upon will be recorded in writing, the written amendment will be signed by the Chief Investigator and by the Principal Investigator and the signed amendment will be appended to this protocol.

Approval / advice of amendments by Ethical Committees or similar body is required prior to their implementation, unless there are overriding safety reasons.

If the change or deviation increases risk to the study population, or adversely affects the validity of the clinical investigation or the subject's rights, full approval / advice must be obtained prior to implementation. For changes that do not involve increased risk or affect the validity of the investigation or the subject's rights, approval / advice may be obtained by expedited review, where applicable.

In some instances, an amendment may require a change to a consent form. The Investigator must receive approval / advice of the revised consent form prior to implementation of the change.

13 REFERENCES

1. Balducci, L., & Ershler, W. B. (2005). Cancer and ageing: a nexus at several levels. Nature Reviews. Cancer, 5 (August), 655–662. doi:10.1038/nrc1675
2. De Angelis, R., Sant, M., Coleman, M. P., Francisci, S., Baili, P., Pierannunzio, D., … Capocaccia, R. (2014). Cancer survival in Europe 1999–2007 by country and age: results of EUROCARE-5—a population-based study. The Lancet Oncology, 15(1), 23–34. doi:10.1016/S1470-2045(13)70546-1
3. Hoffe, S., & Balducci, L. (2012). Cancer and Age: General Considerations. Clinics in Geriatric Medicine, 28, 1–18. doi:10.1016/j.cger.2011.09.001
4. Audisio R.A., van Leeuwen B.L. (2015). Beyond "Age": frailty assessment strategies improve care of older patients with cancer. Annals of Surgical Oncology 22:3774-3775
5. Huisman, M. G., Van Leeuwen, B. L., Ugolini, G., Montroni, I., Spiliotis, J., Stabilini, C., … Audisio, R. a. (2014). "Timed Up & Go": A screening tool for predicting 30-day morbidity in onco-geriatric surgical patients? A multicenter cohort study. PLoS ONE, 9(1). doi:10.1371/journal.pone.0086863
6. Korc-Grodzicki, B., Sun, S. W., Zhou, Q., Iasonos, A., Lu, B., Root, J. C., … Tew, W. P. (2015). Geriatric Assessment as a Predictor of Delirium and Other Outcomes in Elderly Patients With Cancer. Annals of Surgery, 261(6), 1085–1090. doi:10.1097/SLA.0000000000000742
7. Kowdley, G. C., Merchant, N., Richardson, J. P., Somerville, J., Gorospe, M., & Cunningham, S. C. (2012). Cancer Surgery in the Elderly. The Scientific World Journal, 2012, 1–9. doi:10.1100/2012/303852
8. Pearse, R. M., Harrison, D. a, James, P., Watson, D., Hinds, C., Rhodes, A., … Bennett, E. D. (2006). Identification and characterisation of the high-risk surgical population in the United Kingdom. Critical Care (London, England), 10(3), R81. doi:10.1186/cc4928
9. Podsiadlo, D., & Richardson, S. (1991). The timed "Up & Go": a test of basic functional

mobility for frail elderly persons. Journal of the American Geriatrics Society, 39, 142–148. doi:http://www.ncbi.nlm.nih.gov/entrez/query.fcgi?cmd=Retrieve&db=PubMed&dopt=Citation&list_uids=1991946

10. Repetto, L., Comandini, D., & Mammoliti, S. (2001). Life expectancy, comorbidity and quality of life: The treatment equation in the older cancer patients. Critical Reviews in Oncology/Hematology, 37, 147–152. doi:10.1016/S1040-8428(00)00104-9
11. Robinson, T. N., Wu, D. S., Pointer, L., Dunn, C. L., Cleveland, J. C. J., & Moss, M. (2013). Simple frailty score predicts postoperative complications across surgical specialties. American Journal of Surgery, 206(4), 544–550. doi:10.1016/j.amjsurg.2013.03.012
12. Robinson, T. N., Wu, D. S., Pointer, L. F., Dunn, C. L., & Moss, M. (2012). Preoperative cognitive dysfunction is related to adverse postoperative outcomes in the elderly. Journal of the American College of Surgeons, 215(1), 12–18. doi:10.1016/j.jamcollsurg.2012.02.007
13. Shulman, M. a, Myles, P. S., Chan, M. T. V, McIlroy, D. R., Wallace, S., & Ponsford, J. (2015). Measurement of Disability-free Survival after Surgery. Anesthesiology, 122(3), 524–536. doi:10.1097/ALN.0000000000000586
14. Wildiers, H., Heeren, P., Puts, M., Topinkova, E., Janssen-Heijnen, M. L. G., Extermann, M., … Hurria, A. (2014). International Society of Geriatric Oncology Consensus on Geriatric Assessment in Older Patients With Cancer. Journal of Clinical Oncology: Official Journal of the American Society of Clinical Oncology, 32. doi:10.1200/JCO.2013.54.8347
15. Ghignone F, van Leeuwen BL, Montroni I, Huisman MG, Somasundar P, Cheung KL, Audisio RA, Ugolini G; International Society of Geriatric Oncology (SIOG) Surgical Task Force. The assessment and management of older cancer patients: A SIOG surgical task force survey on surgeons' attitudes. Eur J Surg Oncol. 2016 Feb;42(2):297-302. doi: 10.1016/j.ejso.2015.12.004. Epub 2015 Dec 17.
16. Audisio RA. Tailoring surgery to elderly patients with cancer. Br J Surg. 2016 Jan;103(2):e10-1. doi: 10.1002/bjs.9948. Epub 2015 Dec 17. No abstract available. PMID: 26679284
17. Bravo Iñiguez CE, Armstrong KW, Cooper Z, Weissman JS, Ducko CT, Wee JO, Martinez

MP, Bueno R, Jaklitsch MT, Wiener DC. Thirty-Day Mortality After Lobectomy in Elderly Patients Eligible for Lung Cancer Screening. Ann Thorac Surg. 2016 Feb;101(2):541-6. doi: 0.1016/j.athoracsur.2015.08.067. Epub 2015 Oct 23. PMID: 26603020

18. Maillard J, Elia N, Haller CS, Delhumeau C, Walder B. Preoperative and early postoperative quality of life after major surgery - a prospective observational study. Health Qual Life Outcomes. 2015 Feb 4;13:12.
19. Kind P1, Dolan P, Gudex C, Williams A. Variations in population health status: results from a United Kingdom national questionnaire survey. BMJ. 1998 Mar 7;316(7133):736-41.

APPENDIX A: PROTOCOL CHANGES

REASON FOR CHANGES

The protocol has been amended to include patients with moderate severe cognitive impairment into the primary endpoint evaluation, through the use of the proxy version of the EQ-5D-3L questionnaire. Moreover the procedure for Informed Consent obtainment has been better detailed.

Section 5.1. Informed consent pag,12:

Original text

It is the responsibility of the investigator, or a person designated by the investigator, to obtain (if applicable) written informed consent from each individual participating in this study. When applicable, each patient must personally sign and date the latest approved version of the Informed Consent form before any study specific procedures are performed.

Amended text

It is the responsibility of the investigator, or a person designated by the investigator, to obtain (if applicable) written informed consent from each individual participating in this study. When applicable, each patient/**health care proxy** must ~~personally~~ sign and date the latest approved version of the Informed Consent form before any study specific procedures are performed.

Section 5.3.1. Baseline evaluation (CRF B) pag,13:

<u>Original text</u>

[…]

- Self reported Quality of life (EQ-5D-3L). This test will not be administered to patients with moderate severe cognitive impairment (Mini Cog <3)

<u>Amended text</u>

[…]

- ~~Self reported~~ Quality of life (EQ-5D-3L, Self or Proxy-1 version). ~~This test will not be administered to patients with moderate severe cognitive impairment (Mini Cog <3)~~
