# Supplementary material for: Correction to: Quality of Life in Older Adults After Major Cancer Surgery: The GOSAFE International Study
Source: J Natl Cancer Inst. 2024 Apr 29;116(6):996. doi: 10.1093/jnci/djae092 (PMC11160945; doi:10.1093/jnci/djae092)
Supplement: djae092_Supplementary_Data [file djae092_supplementary_data.pdf]

Supplementary Table 1. Recruiting international centers

| Institution | Department | City | Country | Activation date |
|---|---|---|---|---|
| Ospedale "per gli Infermi" AUSL Romagna (Coordinating Center) | U.O. Chirurgia Generale | Faenza (RA) | Italy | 15-Feb-17 |
| Ospedale "GB. Morgagni-L. Pierantoni" AUSL Romagna | U.O. Chirurgia Generale e Terapie Oncologiche Avanzate | Forlì (FC) | Italy | 15-Feb-17 |
| Ospedale "Ceccarini", AUSL Romagna | U.O. Chirurgia Generale | Riccione (RN) | Italy | 09-May-17 |
| AUSLPiacenza, PO Piacenza | U.O. Chirurgia generale | Piacenza | Italy | 11-July-17 |
| Humanitas Clinical and Research Center | Division of Colon and Rectal Surgery, | Rozzano (MI) | Italy | 14-July-17 |
| Ospedale "S. Matteo degli Infermi" AUSL Umbria-2 | General, Minimally Invasive and Robotic Surgery | Spoleto (PG) | Italy | 26-July-17 |
| Brigham and Women's Hospital | Thoracic Surgery | Boston (MA) | USA | 11-Oct-17 |
| Clinica S. Rita | Department of Colorectal Surgery | Vercelli | Italy | 18-Oct-17 |
| Istituto Tumori Giovanni Paolo II IRCCS | Department of Surgical Oncology | Bari | Italy | 22-Nov-17 |
| University of Pennsylvania, Perelman School of Medicine | Department of Surgery | Philadelphia (PA) | USA | 22-Nov-17 |
| University Medical Center Groningen | Department of Surgical Oncology | Groningen | Netherlands | 30-Nov-17 |
| ASST Grande Ospedale Metropolitano Niguarda | Chirurgia generale Oncologia e Mininvasiva | Milan | Italy | 29-Nov-17 |
| Jagiellonian University Medical College | Department of General, Oncologic and Geriatric Surgery | Krakow | Poland | 12-Dec-17 |
| Ospedale Policlinico S. Martino IRCCS | OU General and Oncologic Surgery | Genova | Italy | 06-Dec-17 |
| Oslo University Hospital | Department of Surgery | Oslo | Norway | 12-Dec-17 |
| ASST Monza - Ospedale di Desio | General and Emergency Surgery | Desio (MB) | Italy | 18-Dec-17 |
| Aristotle University of Thessaloniki, Medical School | 4th Surgical Department | Thessaloniki | Greece | 23-Jan-18 |
| Hospital Universitario y Politécnico La Fe | General and Digestive surgery | Valencia | Spain | 09-Feb-18 |
| Roger William Medical Centre Providence | Surgical Oncology | Providence (RI) | USA | 27-Feb-18 |
| Sapienza University of Rome, Sant'Andrea University Hospital | Emergency Surgery Unit | Rome | Italy | 27-Mar-18 |
| Hospital Sao Francisco Xavier | General Surgery | Lisbon | Portugal | 04-Apr-18 |
| Rabin Medical Center | Department of Geriatrics | Tel Aviv | Israel | 26-Apr-18 |
| Ospedale Policlinico S. Martino IRCCS | Department of Surgical Sciences and Integrated Diagnostics (DISC) | Genova | Italy | 14-May-18 |
| Hospital General Universitario de Elche, Universidad Miguel Hernández | Colorectal & Gastrointestinal Department | Alicante | Spain | 21-May-18 |
| Manchester Royal Infirmary, University of Manchester | HPB Unit | Manchester | UK | 16-July-18 |
| Cleveland Clinic Foundation | Department of Colorectal Surgery | Weston (FL) | USA | 11-Jan-19 |

Supplementary Table 2. Functional assessment indicators

| Test | Acronym | Range of possible scores | Frailty indicator threshold | Purpose |
|---|---|---|---|---|
| EQ 5D-3L | EQ 5D-3L Index | 0-1 | Not applicable | Evaluation of QoL assessing patient's mobility, self-care, usual activities, pain and anxiety, includes a visual scale |
| EQ 5D-3L | EQ 5D-3L VAS | 0-100 | Not applicable | |
| Eastern Collaborative Oncology Group Performance Status | ECOG PS | 0-4 | ≥1 | Evaluation of cancer burden on functional status |
| Katz Activities of Daily Living | ADL | 0-6 | <5 | Evaluation of functional independence |
| Mini-Cog | Mini-Cog | 0-5 | ≤2 | Detection of cognitive impairment in older adults therefore suitable for a more thorough evaluation. |
| Flemish version of the Triage Risk Screening Test | fTRST | 0-6 | ≥2 | Detection of hospitalized geriatric patients at risk for frailty |
| Timed Up & Go Test | TUG | Not applicable | ≥20 sec | Three-meters walking test to evaluate functional status |
| Geriatric 8 | G8 | 0-17 | ≤14 | Detection of onco-geriatric patients who may benefit from comprehensive geriatric assessment |
| Nutritional Risk Screening | NRS | Normal to severely impaired nutritional status | Moderately to severely impaired | Evaluation of nutritional status taking into account BMI, weight loss and food intake |
| American Society of Anesthesiology score | ASA | 1-5 | Not applicable | Evaluation of preoperative general clinical condition and estimation of anesthesiologic risk |
| Charlson Age Comorbidity Index | CACI | 0-42 | ≥6 | Evaluation of cumulative burden of patient's comorbidities |

Supplementary Table 3. Type of surgical procedures performed

| Procedure | Frequency |
|---|---|
| Colectomy (left-right-subtotal) | 434 |
| Low anterior resection of the rectum | 144 |
| Abdominoperineal resection | 44 |
| Small bowel resection | 5 |
| | |
| Adrenalectomy | 4 |
| Nephrectomy | 5 |
| Cystectomy | 3 |
| Ureter resection | 1 |
| Prostatectomy | 4 |
| | |
| Hepatectomy (segmental-lobectomy) | 48 |
| Hepatectomy (wedge) | 11 |
| Common bile duct resection | 3 |
| | |
| Lung lobectomy | 29 |
| Lung wedge resection | 3 |
| Chest wall resection | 2 |
| | |
| Gastrectomy total | 25 |
| Gastrectomy subtotal | 67 |
| Esophagectomy | 12 |
| | |
| Whipple | 35 |
| Pancreatectomy distal | 4 |
| Splenectomy | 2 |
| | |
| Sarcoma excision | 5 |
| | |
| Other | 52 |
| | |
| Total | 942 |

Supplementary Table 4. Postoperative complications

| Complications | ≤30 days | | 31-90 days | | 91-180 days | | 0-180 days | |
|---|---|---|---|---|---|---|---|---|
| | CD*I-CDII n (%) | CDIII-CDIV n (%) | CDI-CDII n (%) | CDIII-CDIV n (%) | CDI-CDII n (%) | CDIII-CDIV n (%) | CDI-CDII n (%) | CDIII-CDIV n (%) |
| Patients with at least one complication (CD III-IV) | / | 128 (13.5) | / | 65 (6.9) | / | 52 (5.5) | / | 176 (18.7) |
| Patients with at least one complication (CD I-IV) | 370 (39.2) | | 212 (22.5) | | 210 (22.2) | | 494 (52.4) | |
| Respiratory | 53 (5.6) | 34 (3.6) | 24 (2.6) | 13 (1.4) | 26 (2.9) | 15 (1.7) | 69 (7.7) | 45 (5.0) |
| Cardiac | 35 (3.7) | 20 (2.1) | 14 (1.5) | 13 (1.4) | 16 (1.8) | 8 (0.9) | 44 (4.9) | 37 (4.1) |
| Renal | 54 (5.7) | 7 (2.1) | 15 (1.5) | 3 (1.4) | 10 (1.8) | 2 (0.9) | 66 (4.9) | 12 (4.1) |
| Neurological | 25 (2.7) | 1 (0.1) | 8 (0.9) | 6 (0.7) | 9 (1.0) | 2 (0.2) | 38 (4.2) | 8 (0.9) |
| Nutritional | 24 (2.5) | 4 (0.4) | 11 (1.2) | 1 (0.1) | 10 (1.1) | 3 (0.3) | 36 (4.0) | 7 (0.8) |
| Pressure sores | 2 (0.2) | 2 (0.2) | 1 (1.0) | 0 (0.0) | 0 (0.0) | 1 (0.1) | 2 (0.2) | 3 (0.3) |
| Pain | 6 (0.6) | 2 (0.2) | 1 (0.1) | 2 (0.2) | 4 (0.4) | 2 (0.2) | 10 (1.1) | 3 (0.3) |
| Delirium | 11 (1.2) | 0 (0.0) | 3 (0.3) | 0 (0.0) | 0 (0.0) | 0 (0.0) | 13 (1.5) | 0 (0.0) |
| Wound | 44 (4.7) | 14 (1.5) | 29 (3.2) | 6 (0.7) | 32 (3.6) | 1 (0.1) | 82 (9.2) | 16 (1.8) |
| Gastrointestinal | 65 (6.9) | 52 (5.5) | 46 (5.0) | 19 (2.1) | 38 (4.2) | 17 (1.9) | 110 (12.3) | 73 (8.1) |
| Other complication | 73 (7.7) | 31 (3.3) | 41 (4.5) | 15 (1.6) | 39 (4.4) | 11 (1.2) | 153 (17.1) | 57 (6.4) |

*CD: Clavien-Dindo classification complication grade

Supplementary Figure 1. The EQ-5D-3L.

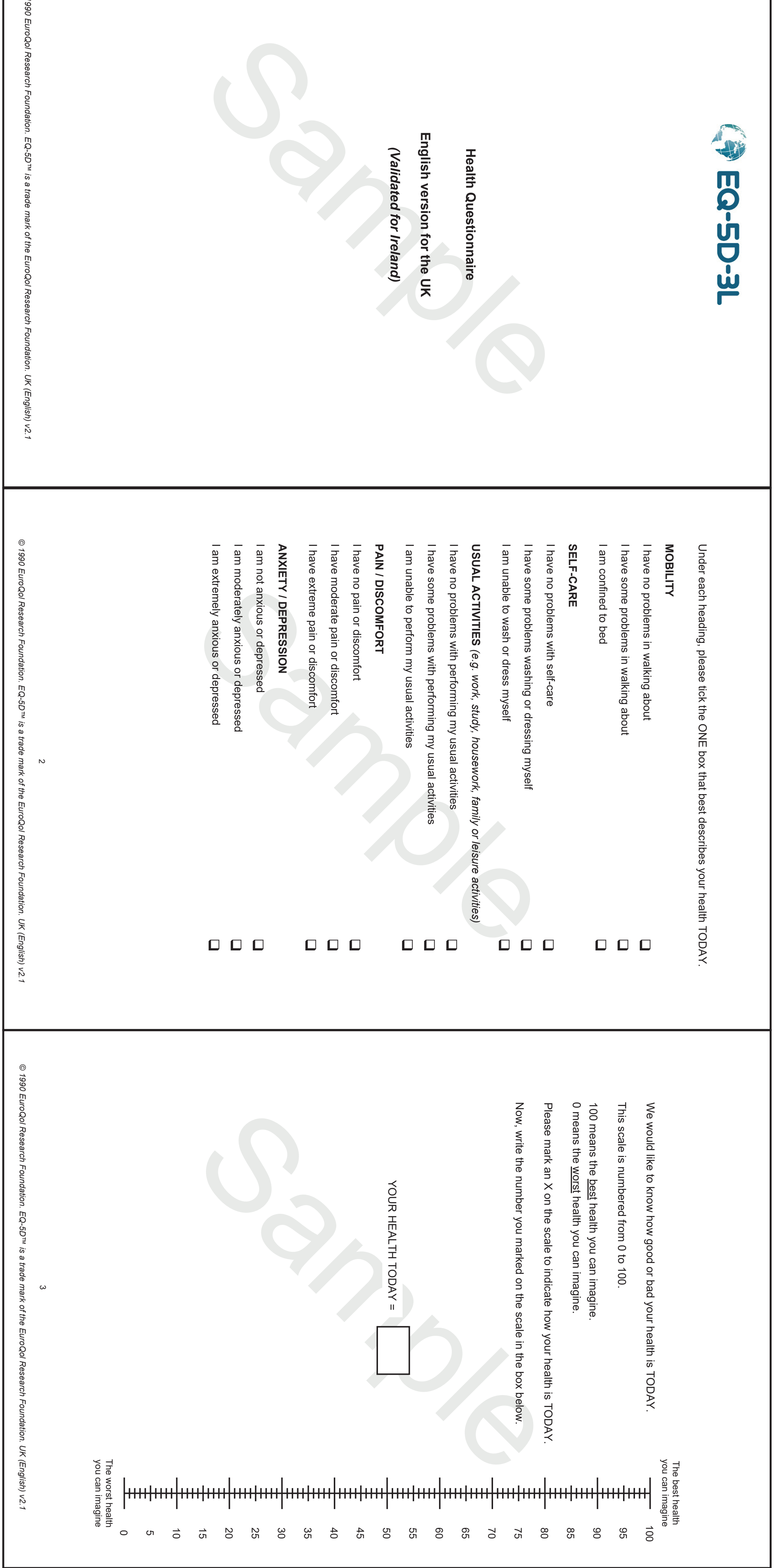

EQ-5D-3L

**Health Questionnaire**

**English version for the UK**

***(Validated for Ireland)***

© 1990 EuroQol Research Foundation. EQ-5D™ is a trade mark of the EuroQol Research Foundation. UK (English) v2.1

Under each heading, please tick the ONE box that best describes your health TODAY.

**MOBILITY**

- I have no problems in walking about ☐
- I have some problems in walking about ☐
- I am confined to bed ☐

**SELF-CARE**

- I have no problems with self-care ☐
- I have some problems washing or dressing myself ☐
- I am unable to wash or dress myself ☐

**USUAL ACTIVITIES** *(e.g. work, study, housework, family or leisure activities)*

- I have no problems with performing my usual activities ☐
- I have some problems with performing my usual activities ☐
- I am unable to perform my usual activities ☐

**PAIN / DISCOMFORT**

- I have no pain or discomfort ☐
- I have moderate pain or discomfort ☐
- I have extreme pain or discomfort ☐

**ANXIETY / DEPRESSION**

- I am not anxious or depressed ☐
- I am moderately anxious or depressed ☐
- I am extremely anxious or depressed ☐

2

© 1990 EuroQol Research Foundation. EQ-5D™ is a trade mark of the EuroQol Research Foundation. UK (English) v2.1

- We would like to know how good or bad your health is TODAY.
- This scale is numbered from 0 to 100.
- 100 means the best health you can imagine.
- 0 means the worst health you can imagine.
- Please mark an X on the scale to indicate how your health is TODAY.
- Now, write the number you marked on the scale in the box below.

YOUR HEALTH TODAY = ☐

The best health you can imagine

100, 95, 90, 85, 80, 75, 70, 65, 60, 55, 50, 45, 40, 35, 30, 25, 20, 15, 10, 5, 0

The worst health you can imagine

3

© 1990 EuroQol Research Foundation. EQ-5D™ is a trade mark of the EuroQol Research Foundation. UK (English) v2.1

Supplementary Methods: study protocol

esso
THE EUROPEAN SOCIETY OF SURGICAL ONCOLOGY

SIOG
INTERNATIONAL SOCIETY OF GERIATRIC ONCOLOGY

# GO SAFE Study

# Geriatric Oncology Surgical Assessment and Functional rEcovery after Surgery

## An international prospective audit to evaluate postoperative functional outcomes and quality of life after cancer surgery in geriatric patients

**Date and Version: 15/03/2017 - Amendment 1.0**

## KEY TRIAL CONTACTS

| | |
|---|---|
| **Chief Investigator:** | **Prof. Giampaolo Ugolini**<br>Alma Mater University of Bologna<br>Ospedale degli Infermi, Faenza (RA), IT<br>General Surgery Unit<br>Telephone +39 349 4347576<br>e-mail address: g.ugolini@unibo.it |
| **Co-Investigators:** | **Dr. Barbara Van Leeuwen**<br>Dept. of Surgical Oncology<br>UMCG, Groningen, NL |
| **Sponsor/Promoter:** | **AUSL della Romagna**<br>Via de Gasperi 8<br>48121 Ravenna |
| **Study management:** | **Unità di Biostatistica e Sperimentazioni Cliniche**<br>Istituto Scientifico Romagnolo per lo Studio e la Cura dei Tumori (IRST) s.r.l IRCCS<br>Via Piero Maroncelli, 40/42<br>47014 Meldola (FC)<br>Telephone + 390544285813 Fax +390544285330<br>e-mail address: cc.ubsc@irst.emr.it |
| **Statistician:** | **Dr. Oriana Nanni**<br>Istituto Scientifico Romagnolo per lo Studio e la Cura dei Tumori (IRST) s.r.l IRCCS<br>Via Piero Maroncelli, 40/42<br>47014 Meldola (FC)<br>e-mail address: oriana.nanni@irst.emr.it |

**Steering committee:**

**Riccardo Audisio**
Dept. of Surgical Oncology - St Helens Hospital Liverpool, UK

**Nicola De Liguori Carino**
HBP Surgery, Manchester Royal Infirmary, UK

**Giorgio Ercolani**
Alma Mater University of Bologna - General Surgery Unit - Ospedale Morgagni, Forlì, IT

**Nadav Haim**
Colorectal surgery Sheba Medical Center Ramat Gan, HaMerkaz, Israel

**Michael Jaklitsch**
Dept of Thoracic Surgery - Brigham and Women's Hospital, Boston, MA, USA

**Isacco Montroni**
General Surgery Unit - Ospedale degli Infermi, Faenza, IT

**Siri Rostoft**
Dept. of Geriatric Oncology, Oslo University Hospital, NW

**Ponnandai Somasundar**
Dept. of Surgical Oncology, Roger Williams Medical Center Providence - Boston University, RI, USA

**Promoted by SIOG surgical task force and ESSO**

**Confidentiality Statement**
This document contains confidential information that must not be disclosed to anyone other than the Sponsor/Promoter, the Investigator’s Team, IRST IRCCS, regulatory authorities, and members of the Ethics Committee.

**Protocol approval and Investigator agreement**

**Geriatric Oncology Surgical Assessment and Functional rEcovery after Surgery**

The undersigned agree and confirm that:

The following protocol has been agreed and accepted and the Chief Investigator agrees to conduct the trial in compliance with the approved protocol and will adhere to the principles outlined in ICH GCP guidelines, Sponsor/Promoter SOP's and other regulatory requirements as amended.

The confidential information contained in this document will not be used for any other purpose other than the evaluation or conduct of the clinical investigation without the prior written consent of the Sponsor/Promoter.

The findings of the study will be made publically available through publication or other dissemination tools without any unnecessary delay and that an honest accurate and transparent account of the study will be given; and any discrepancies from the study as planned in this protocol will be explained.

Giampaolo Ugolini

| | | |
|---|---|---|
| Chief Investigator | Signature | Date |

Oriana Nanni

| | | |
|---|---|---|
| Trial Statistician | Signature | Date |

By signing this document I am confirming that I have read the protocol for the above study and I agree to conduct the study in compliance with the protocol and ICH GCP.

___________________

| | | |
|---|---|---|
| Principal Investigator | Signature | Date |

**ABBREVIATIONS**

| | |
|---|---|
| AE | Adverse event |
| AR | Adverse reaction |
| CC | Coordinating Center |
| CI | Chief Investigator |
| CRA | Clinical Research Associate (Monitor) |
| CRF | Case Report Form |
| CRO | Contract Research Organisation |
| CT | Clinical Trials |
| CTC | Common toxicity criteria |
| ECOG | Performance status (Eastern Cooperative Oncology Group, ECOG Scale) |
| FR | Functional recovery |
| GCP | Good Clinical Practice |
| IB | Investigators Brochure |
| ICF | Informed Consent Form |
| ICH | International Conference of Harmonisation |
| IDMC | Indipendent Data Monitoring Committee |
| IEC | Independent Ethics Committee |
| IMP | Investigational Medicinal Products |
| IRB | Independent Review Board |
| PI | Principal Investigator |
| QoL | Quality of Life |
| RECIST | Response Evaluation Criteria In Solid Tumors |
| SAE | Serious Adverse Event |
| SAR | Serious Adverse Reaction |
| SOP | Standard Operating Procedure |
| SUSAR | Suspected Unexpected Serious Adverse Reactions |
| WHO | World Health Organization |

# TABLE OF CONTENTS

KEY TRIAL CONTACTS ........ 2
STEERING COMMITTEE: ........ 3
1. BACKGROUND ........ 8
2. RATIONALE ........ 9
3. AIM OF THE STUDY ........ 9
3.1 PRIMARY OBJECTIVE ........ 10
3.2 SECONDARY OBJECTIVES ........ 10
4. STUDY PROTOCOL ........ 10
4.1 CENTRES AND INVESTIGATORS ........ 10
4.2 STUDY DESIGN ........ 11
4.3 STUDY POPULATION ........ 11
4.3.1 INCLUSION CRITERIA ........ 11
4.3.2 EXCLUSION CRITERIA ........ 11
4.4 LOCAL APPROVALS ........ 12
5. PROCEDURES AND DATA COLLECTION ........ 12
5.1 INFORMED CONSENT ........ 12
5.2 REGISTRATION (CRF A) ........ 12
5.3 DATA COLLECTION ........ 13
5.3.1 BASELINE EVALUATION (CRF B) ........ 13
5.3.2 OPERATIVE DETAILS AND EARLY POSTOPERATIVE OUTCOME (CRF C) ........ 14
5.3.3 FOLLOW UP (CRF 3M-6M) ........ 15
5.4 STUDY PLAN FLOWSHEET AND CRF COMPLETION TIMES ........ 16
5.5 CONFIDENTIALITY ........ 16
5.6 DATA QUALITY ASSURANCE ........ 17
6 STATISTICAL CONSIDERATIONS ........ 17
6.1 DATA ANALYSIS ........ 17
6.2 SAMPLE SIZE ........ 17
6.3 STUDY DURATION ........ 18
7 WITHDRAWAL OF PATIENTS FROM THE STUDY ........ 18

**8 ETHICAL ASPECTS ....................................................................................................18**

8.1 LOCAL REGULATIONS/DECLARATION OF HELSINKI ........................................................ 18

8.2 INDEPENDENT ETHICAL COMMITTEE ........................................................................ 18

8.3 INFORMED CONSENT ............................................................................................. 19

8.4 PATIENT DATA PROTECTION ................................................................................... 19

**9 ADMINISTRATIVE REGULATIONS ...............................................................................19**

9.1 CURRICULUM VITAE .............................................................................................. 20

9.2 SECRECY AGREEMENT ........................................................................................... 20

9.3 FINANCIAL ARRANGEMENTS ................................................................................... 20

**10 OWNERSHIP OF THE DATA AND USE OF THE STUDY RESULTS.....................................20**

**11 PUBLICATION POLICY AND AUTHORSHIP....................................................................20**

**12 PROTOCOL AMENDMENTS .........................................................................................21**

**13 REFERENCES ..............................................................................................................22**

## 1. BACKGROUND

Progressive aging of the world population has become one of the most significant challenges for national health care systems. With aging, the incidence and prevalence of cancer increases: it has been estimated that in 2020 more than 60% of all malignancies will occur in patients aged 70-year and older. At the same time, progress in medical knowledge has determined an extremely positive impact in clinical practice. In particular, improvements in perioperative care, surgical minimally invasive techniques and the introduction of multimodal treatment have made surgery feasible for a higher number of patients. Nevertheless, several studies show that senior adults affected by cancer are often treated sub-optimally, above all in the surgical field.

It is well known that onco-geriatric patients are at higher risk of developing postoperative complications because they are often affected by multiple comorbidities. It has been reported that up to 80% of elderly patients might experience a surgical complication. Thus, after major surgery, patients may be at risk of both developing postoperative complications, and suffering major discomfort that can negatively affect postoperative quality of life.

The vast majority of research studies are focused on short-term outcomes and do not explore long- term disability or postoperative quality of life.

Onco-geriatric patients represent a challenge for surgical oncologists because, despite the evidence that comorbidities are often responsible for poor postoperative outcomes, patients' selection has not been completely standardized yet. Preoperative assessment of the functional status is fundamental to identify fit, vulnerable and frail individuals in order to avoid under- or over-treatment. Functional recovery has been shown to be of critical value in the elderly population since restoration/conservation of independence is probably the most important end-point for senior adults. Individualization of elderly cancer-patients care is closely related with the possibility of preserving their functional capacity. We could conclude that for elderly patients, perhaps more than anyone else, "quality" is more important than "quantity" of life.

## 2. RATIONALE

We aim to improve outcomes of onco-geriatric patients' surgical management. Our research project will focus on quality of life and functional recovery after surgery. The most important expected result will be the collection of data that clinicians will be able to exploit in the management of frail and 'pre-frail' patients with the potential to reduce disparities in elderly patient care. In addition, the 'multidisciplinary work ethic' in the management of this specific group of patients, regardless for their primary condition, will be extensively promoted to determine small but clinically important incremental improvements in elderly care.

We need to conduct the GO SAFE study for several reasons:

- There is a dramatic lack of knowledge on elderly cancer surgical patients
- Although survival is commonly reported after surgery, quality of life (QoL) and functional recovery (FR), including nutritional status, are rarely measured
- To promote the practice of a multidisciplinary management of elderly cancer patients
- To understand how frailty, comorbidities and malnourishment are associated with early and long-term clinical outcomes after surgery in elderly cancer patients
- To obtain prospective data to assist clinicians in tailoring the care, avoiding under/over-treatment
- To identify new strategies to improve functional outcomes (as cardiorespiratory/nutritional prehabilitation)

To identify areas warranting further research studies and surgical audit in the older adults cancer population

## 3. AIM OF THE STUDY

GO SAFE study is a prospective international collaborative high-quality registry aiming to gain knowledge about postoperative outcomes in older cancer patients with a particular emphasis on QoL and FR. The target is to obtain meaningful data to assist clinicians in tailoring the care,

avoiding under/over-treatment, providing robust data to identify new strategies to improve functional outcomes in older cancer patients.

### *3.1 Primary Objective*

To evaluate the effects of surgery on patients' life perception by comparing pre- and post-operative QoL in elderly patients undergoing major surgery for solid malignancies using a self-reported Quality of Life assessment tool (EQ 5D-3L)

### *3.2 Secondary Objectives*

- To evaluate FR in terms of nutritional status, restoration of daily activities (ADL) and cognitive status (Mini-Cog)
- To evaluate 3 and 6 months postoperative morbidity and mortality
- To obtain prognostic factors for postoperative functional recovery which will assist in the treatment planning /intervention of future elderly patients who are offered surgery for cancer
- To identify variables affecting postoperative quality of life

## 4. STUDY PROTOCOL

### *4.1 Centres and Investigators*

We aim to involve in the study as many centers as possible. All surgical units performing cancer surgery in elderly patients are invited to participate. Participating investigators will be surgical oncologists. Each center will require approval from local Institutional Review Board and/or the Ethic Committee before starting to enroll patients.

Investigators will be responsible to obtain a written informed consent from each eligible patient in according to the local IRB regulation, ahead of surgery (please see note below in case of demented patients). Every center shall commit to send clinical data after the 6-month follow up.

Periodic evaluation of patient data entry, centers' activity, and cohesion of the centers will be shared among the investigators.

### *4.2 Study Design*

GO SAFE is a multicenter international observational prospective cohort study. The study is non-for-profit. Recruiting centers will collect data prospectively. Recruited patients will be followed for 6 months after their surgery. The original treatment plan, as designed by each individual recruiting centre, will not be altered or affected by the study inclusion.

### *4.3 Study Population*

Centers should ensure that they would make every possible effort to include all consecutive eligible patients during the study period and provide completeness of data entry to ensure a 'real-life' study.

#### *4.3.1 Inclusion Criteria*

1. All consecutive patients, both gender, aged ≥70
2. Patients affected by solid malignancy
3. Patients undergoing elective major surgical procedures with curative or palliative intent (all major procedures including any resection, for any cancer, via any operative approach, open, laparoscopic, robotic, etc…)
4. Informed consent obtainment

#### *4.3.2 Exclusion criteria*

1. Patients undergoing emergent/urgent surgical procedures
2. Planned hospital stay less than 48 hours

### *4.4 Local approvals*

Inclusion in the study does not imply any deviation from the current standard of practice, and no change is expected to the perioperative treatment at any point. Patients will be only asked to complete simple screening/assessment tests: for this reason this study should be registered as a prospective observational study at each participating hospital IRB. It is the responsibility of the local team to ensure that regulatory process is completed for its hospital. Participating centres will be asked to confirm that they have gained formal approval and to provide an Identification Number.

## 5. PROCEDURES AND DATA COLLECTION

### *5.1 Informed Consent*

It is the responsibility of the investigator, or a person designated by the investigator, to obtain (if applicable) written informed consent from each individual participating in this study. When applicable, each patient/health care proxy must sign and date the latest approved version of the Informed Consent form before any study specific procedures are performed.

Patients must receive an explanation that they are completely free to refuse to enter in this study and to withdraw from it at any time and for any reason without prejudice to future care, and with no obligation to give the reason for withdrawal. The original signed form will be retained at the study site. A copy of the informed consent form will be delivered to the patient. A form for obtaining written informed consent for this observational study will be provided.

### *5.2 Registration (CRF A)*

All patients for whom eligibility criteria have been verified will be registered by each participating center in the eCRF.

The following data will be collected at registration:

- Patient’s Date of birth
- Patient’s Gender
- Date of Informed consent
- Date of registration

- Center informations

### *5.3 Data Collection*

Clinical reporting forms (CRFs) have been designed to be completed along the normal daily-life practice, trying to minimize the 'extra work' for local investigators. Tests, carried out at base-line and follow-up evaluation, could be easily completed by surgical trainees, medical students and nurses adequately trained. Surgical data analysis, including detection of postoperative complications, should require the supervision of an attending/consultant surgeon.

CRFs are to be completed through use of an EDC system. Sites will have access to a manual for appropriate CRF completion. All CRFs should be completed by designated, trained site staff. CRFs should be reviewed and electronically signed and dated by the investigator or a designee.

If a correction is required for an CRF, the EDC system will create an electronic audit trail.

Participants must maintain quality of their database and update their database. Access to the raw data will be according to agreement of both parties. Each center must keep a file of all consecutive patients that have been entered in the database for random quality monitoring.

#### *5.3.1 Baseline evaluation (CRF B)*

For every eligible patient, demographic data will be collected followed by a fast preoperative functional assessment including:

- Charlson Comorbidity Index
- "Timed Up and Go" test
- Nutritional Risk Screening (NRS)
- American Society of Anesthesiology (ASA) score
- ECOG Performance Status
- G8 geriatric screening tool
- Mini-Cog
- Activities of Daily Living (ADL)
- Quality of Life (EQ 5D-3L, Self or Proxy-1 version).

- History of delirium during illness or hospital admission
- History of Smoking
- History of falls in the 6 months prior to the operation
- Living situation
- Lab's (Albumin, Hemoglobin, Creatinin)
- Polipharmacotherapy (total number of medications)
- Preoperative chemotherapy/radiation therapy
- Involvement of geriatric specialist in preoperative care

#### ***5.3.2 Operative details and early postoperative outcome (CRF C)***

Data regarding surgical procedures and perioperative measures will be collected. Complications will be reported and graded according to Clavien-Dindo Classification.

- Cancer site
- Surgical Procedure Category: Ortho, Gyn, Breast, Upper-GI, Colorectal, HBP, Peritoneum, Thoracic (esophagus), Head & Neck, Urology
- Type of procedure (describe)
- Type of anesthesia (General, Spinal, Epidural)
- Type of surgery (palliative, curative)
- Duration of anaesthesia (min)
- Surgical approach (Open/Laparoscopic/Robotic….)
- Need of ICU stay (Y/N; n. days)
- Perioperative blood transfusions (Units of Packed RBC's) within the surgical admission
- Postoperative length of stay (days in surgical unit)
- Patient discharged to same preoperative setting
- Patient transferred to Medicine/Rehabilitation facility
- Tumor stage (TNM/Stage)

- Involvement of geriatric specialist in postoperative care
- 30 day morbidity (Clavien-Dindo)
- 30 day mortality

#### *5.3.3 Follow up (CRF 3M-6M)*

Three- and six-month follow up data will be collected after surgery within a range of 2 weeks from the due date.

| Data | 3 months | 6 months |
|---|---|---|
| • Morbidity (Clavien-Dindo) | X | X |
| • Mortality | X | X |
| • Living situation | X | X |
| • Weight | X | X |
| • Nutritional Screenig | X | X |
| • “Timed Up and Go” test | X | X |
| • Mini-Cog | X | X |
| • ECOG Performance Status | X | X |
| • ADL | X | X |
| • Self-reported Quality of Life (EQ 5D-3L) | X | X |
| • Postoperative Chemotherapy | X | X |
| • Postoperative Radiation Therapy | X | X |
| • Rehabilitation program | X | X |
| • Nutritional supplement | X | X |

| | | |
|---|---|---|
| • Involvement of a geriatric specialist | X | X |

Local investigators should be also proactive in identifying postoperative events. For example they may review patients notes during admission and before discharge, as well as they could review hospital and outpatient clinic systems to check for readmission and/or other unplanned events.

### 5.4 Study plan flowsheet and CRF completion times

| | |
|---|---|
| REGISTRATION | CRF A |
| PREOPERATIVE ASSESSMENT (BASELINE EVALUATION) ↓ | CRF B |
| OPERATIVE DETAILS EARLY POSTOPERATIVE OUTCOME (1 month) ↓ | CRF C |
| FOLLOW UP 3 months | CRF 3M |
| 6 months | CRF 6M |

### 5.5 Confidentiality

Personal patients' data will not be shared to anyone outside of the research team. The information collected in this research project will be kept private. All patient information will be anonymized. The database used is certified, highly secured and is stored in a encrypted server that meets all the requirements for data-safety and privacy set by international law.

### *5.6 Data quality assurance*

- Medical review with investigators
- CRF quality check, query firing, data cleaning
- Early feedback with local research team via teleconferences

## 6 STATISTICAL CONSIDERATIONS

### *6.1 Data analysis*

The Full Analysis Set (FAS) consists of all registered patients.

The primary endpoint will be measured for all registered subjects who fulfill preoperative and postoperative EQ VAS. Demographic and baseline patient characteristics will be summarized for all patients in the FAS. Continuous-scaled variables (e.g., age) will be summarized with means, medians, standard deviations, quartiles, and minimum and maximum values. Categorical variables (e.g., sex) will be summarized using patient counts and percentages. Study endpoints and variables will be evaluated using descriptive statistics, and the key figures of the distributions will be presented in tables. Univariate analyses will allow for a first overview of potentially influential factors.

Multiple linear regression models will be performed in order to evaluate predictors of functional recovery at 3 months and 6 months after surgery.

Exploratory subgroup analyses will be performed. Missing values will be replaced and estimated using multiple imputations. Furthermore, sensitivity analysis will be executed using complete-case analysis.

### *6.2 Sample size*

A sample size of 265 patients who completed pre and postoperative EQ VAS questionnaires will have a 90% power to detect an effect size of 0,2 between pre and post surgery ,using a paired t-test with a 0,05 two sided significance level.

Given a potential loss to follow-up (about 10%), uncompleted questionnaires (about 10%) and postoperative mortality (about 15%), the sample size will be increased to 350-400 patients (see

ref 18 and 19).

### *6.3 Study duration*

Enrollment period: 24 months

Follow- up: 6 months

Data analysis : 6 months

Total duration of the study: 36 months

## 7 WITHDRAWAL OF PATIENTS FROM THE STUDY

Patients have the right to withdraw at any time for any reason during their participation in this observational study.

## 8 ETHICAL ASPECTS

### *8.1 Local regulations/Declaration of Helsinki*

The responsible Investigator will ensure that this study is conducted in compliance with the protocol, following the instructions and procedures described, adhering to the principles of Good Clinical Practice ICH Tripartite Guideline (December 2000) and in accordance with the principles laid down by the 18th World Medical Assembly (Helsinki, 1964 and further amendments) or with the laws and regulations of the country in which the research is conducted, whichever affords the greater protection to the individual.

### *8.2 Independent Ethical Committee*

The protocol, informed consent and any accompanying material provided to the patient will be submitted by the investigator to an Independent Ethical Committee for review. Approval from the committee must be obtained before starting the study. Any modifications made to the protocol, informed consent or material provided to the patient after receipt of the Ethics Committee approval must also be submitted by the investigator to the Committee in accordance with local procedures and regulatory requirements. The Independent Ethical

Committee approval report must contain details of the trial (title, protocol number and version), documents evaluated (protocol, informed consent, accompanying material) and the date of the approval.

### *8.3 Informed Consent*

It is the responsibility of the Investigator to obtain written informed consent from each subject prior to entering the trial or, where relevant, prior to evaluating the subject's suitability for the study.

The informed consent document used by the Investigator for obtaining the subject's informed consent must be reviewed and approved by the Ethical Committee.

A copy of the patient's signed written consent will be kept by the center in the proper section of the Investigator Site File.

### *8.4 Patient data protection*

The Informed Consent Form will incorporate wording that complies with relevant data protection and privacy legislation. In agreement with this wording, patients will authorize the collection, use and disclosure of their study data and samples by the Investigator and by those persons who need that information for the purposes of the study.

The Informed Consent Form will explain that the study data will be stored in a computer data base, maintaining confidentiality in accordance with national data legislation.

The Informed Consent Form will explain that the samples obtained by patients will be anonymized and stored in accordance with national data legislation.

The Informed Consent Form will also explain that for data verification purposes, authorized representatives of Sponsor/Promoter, a regulatory authority, an Ethics Committee may require direct access to parts of the hospital or practice records relevant to the study, including patients' medical history.

## 9 ADMINISTRATIVE REGULATIONS

The Coordinating Center (CC) is responsible for drawing up the final version of the protocol, implementing the CRFs and the electronic database, defining general

organizational procedures and organizing periodic meetings and newsletters. The CC will also undertake the following: support for the preparation of all documents needed for EC submission of the study protocol for each participating center, training of staff assigned to data collection, definition of monitoring procedures.

### *9.1 Curriculum vitae*

An updated copy of the curriculum vitae of each Principal Investigator, duly signed and dated, will be provided to the CC prior to the beginning of the study.

### *9.2 Secrecy agreement*

All goods, materials, information (oral or written) and unpublished documentation provided to the Investigators, including this protocol and the case report forms, shall be considered confidential and may not given or disclosed to third parties.

### *9.3 Financial Arrangements*

This is a non-for-profit study promoted by SIOG surgical task force and ESSO. No registration fee is requested to participate to the GO SAFE study.

No financial reimbursements will be made to participating centers/investigators.

## 10 OWNERSHIP OF THE DATA AND USE OF THE STUDY RESULTS

Participants shall retain the ownership of their own data. Each participating center is responsible for accurate data entry and has access to their data only. No data sharing will be performed with any third party. Personal data will be anonymized and confidential encrypted in a secure place. Professional support for data analysis will be made available.

## 11 PUBLICATION POLICY AND AUTHORSHIP

Clinical results will be published collaboratively. Interim and final analysis will be presented at scientific conferences to ensure visibility.

Data will be published, acknowledging authorship to all the centers giving a substantial contribution, under the name of ”SIOG (International Society of Geriatric Oncology)

surgical task force/ESSO (European Society of Surgical Oncology) GO SAFE study group".

A maximum of 5 investigators from each individual surgical unit will be included as formal co-investigators in this research, and will be PubMed searchable and citable. The output from this research will be published on behalf of the "SIOG (International Society of Geriatric Oncology) surgical task force/ESSO (European Society of Surgical Oncology) GO SAFE study group".

Each hospital may participate with different surgical units (GI, HBP, etc…) and each unit should enrol a minimum number of 20 patients in order to claim authorship.

## 12 PROTOCOL AMENDMENTS

It is specified that the appendices, attached to this protocol and referred to in the main text of this protocol, form an integral part of the protocol.

No changes or amendments to this protocol may be made by the Investigators after the protocol has been agreed to and signed by both parties . Any change agreed upon will be recorded in writing, the written amendment will be signed by the Chief Investigator and by the Principal Investigator and the signed amendment will be appended to this protocol.

Approval / advice of amendments by Ethical Committees or similar body is required prior to their implementation, unless there are overriding safety reasons.

If the change or deviation increases risk to the study population, or adversely affects the validity of the clinical investigation or the subject's rights, full approval / advice must be obtained prior to implementation. For changes that do not involve increased risk or affect the validity of the investigation or the subject's rights, approval / advice may be obtained by expedited review, where applicable.

In some instances, an amendment may require a change to a consent form. The Investigator must receive approval / advice of the revised consent form prior to implementation of the change.

## 13 REFERENCES

1. Balducci, L., & Ershler, W. B. (2005). Cancer and ageing: a nexus at several levels. Nature Reviews. Cancer, 5 (August), 655–662. doi:10.1038/nrc1675
2. De Angelis, R., Sant, M., Coleman, M. P., Francisci, S., Baili, P., Pierannunzio, D., … Capocaccia, R. (2014). Cancer survival in Europe 1999–2007 by country and age: results of EUROCARE-5—a population-based study. The Lancet Oncology, 15(1), 23–34. doi:10.1016/S1470-2045(13)70546-1
3. Hoffe, S., & Balducci, L. (2012). Cancer and Age: General Considerations. Clinics in Geriatric Medicine, 28, 1–18. doi:10.1016/j.cger.2011.09.001
4. Audisio R.A., van Leeuwen B.L. (2015). Beyond "Age": frailty assessment strategies improve care of older patients with cancer. Annals of Surgical Oncology 22:3774-3775
5. Huisman, M. G., Van Leeuwen, B. L., Ugolini, G., Montroni, I., Spiliotis, J., Stabilini, C., … Audisio, R. a. (2014). "Timed Up & Go": A screening tool for predicting 30-day morbidity in onco-geriatric surgical patients? A multicenter cohort study. PLoS ONE, 9(1). doi:10.1371/journal.pone.0086863
6. Korc-Grodzicki, B., Sun, S. W., Zhou, Q., Iasonos, A., Lu, B., Root, J. C., … Tew, W. P. (2015). Geriatric Assessment as a Predictor of Delirium and Other Outcomes in Elderly Patients With Cancer. Annals of Surgery, 261(6), 1085–1090. doi:10.1097/SLA.0000000000000742
7. Kowdley, G. C., Merchant, N., Richardson, J. P., Somerville, J., Gorospe, M., & Cunningham, S. C. (2012). Cancer Surgery in the Elderly. The Scientific World Journal, 2012, 1–9. doi:10.1100/2012/303852
8. Pearse, R. M., Harrison, D. a, James, P., Watson, D., Hinds, C., Rhodes, A., … Bennett, E. D. (2006). Identification and characterisation of the high-risk surgical population in the United Kingdom. Critical Care (London, England), 10(3), R81. doi:10.1186/cc4928
9. Podsiadlo, D., & Richardson, S. (1991). The timed "Up & Go": a test of basic functional

mobility for frail elderly persons. Journal of the American Geriatrics Society, 39, 142–148. doi:http://www.ncbi.nlm.nih.gov/entrez/query.fcgi?cmd=Retrieve&db=PubMed&dopt=Citation&list_uids=1991946

10. Repetto, L., Comandini, D., & Mammoliti, S. (2001). Life expectancy, comorbidity and quality of life: The treatment equation in the older cancer patients. Critical Reviews in Oncology/Hematology, 37, 147–152. doi:10.1016/S1040-8428(00)00104-9
11. Robinson, T. N., Wu, D. S., Pointer, L., Dunn, C. L., Cleveland, J. C. J., & Moss, M. (2013). Simple frailty score predicts postoperative complications across surgical specialties. American Journal of Surgery, 206(4), 544–550. doi:10.1016/j.amjsurg.2013.03.012
12. Robinson, T. N., Wu, D. S., Pointer, L. F., Dunn, C. L., & Moss, M. (2012). Preoperative cognitive dysfunction is related to adverse postoperative outcomes in the elderly. Journal of the American College of Surgeons, 215(1), 12–18. doi:10.1016/j.jamcollsurg.2012.02.007
13. Shulman, M. a, Myles, P. S., Chan, M. T. V, McIlroy, D. R., Wallace, S., & Ponsford, J. (2015). Measurement of Disability-free Survival after Surgery. Anesthesiology, 122(3), 524–536. doi:10.1097/ALN.0000000000000586
14. Wildiers, H., Heeren, P., Puts, M., Topinkova, E., Janssen-Heijnen, M. L. G., Extermann, M., … Hurria, A. (2014). International Society of Geriatric Oncology Consensus on Geriatric Assessment in Older Patients With Cancer. Journal of Clinical Oncology: Official Journal of the American Society of Clinical Oncology, 32. doi:10.1200/JCO.2013.54.8347
15. Ghignone F, van Leeuwen BL, Montroni I, Huisman MG, Somasundar P, Cheung KL, Audisio RA, Ugolini G; International Society of Geriatric Oncology (SIOG) Surgical Task Force. The assessment and management of older cancer patients: A SIOG surgical task force survey on surgeons' attitudes. Eur J Surg Oncol. 2016 Feb;42(2):297-302. doi: 10.1016/j.ejso.2015.12.004. Epub 2015 Dec 17.
16. Audisio RA. Tailoring surgery to elderly patients with cancer. Br J Surg. 2016 Jan;103(2):e10-1. doi: 10.1002/bjs.9948. Epub 2015 Dec 17. No abstract available. PMID: 26679284
17. Bravo Iñiguez CE, Armstrong KW, Cooper Z, Weissman JS, Ducko CT, Wee JO, Martinez

MP, Bueno R, Jaklitsch MT, Wiener DC. Thirty-Day Mortality After Lobectomy in Elderly Patients Eligible for Lung Cancer Screening. Ann Thorac Surg. 2016 Feb;101(2):541-6. doi: 0.1016/j.athoracsur.2015.08.067. Epub 2015 Oct 23. PMID: 26603020

18. Maillard J, Elia N, Haller CS, Delhumeau C, Walder B. Preoperative and early postoperative quality of life after major surgery - a prospective observational study. Health Qual Life Outcomes. 2015 Feb 4;13:12.
19. Kind P1, Dolan P, Gudex C, Williams A. Variations in population health status: results from a United Kingdom national questionnaire survey. BMJ. 1998 Mar 7;316(7133):736-41.

## APPENDIX A: PROTOCOL CHANGES

## REASON FOR CHANGES

The protocol has been amended to include patients with moderate severe cognitive impairment into the primary endpoint evaluation, through the use of the proxy version of the EQ-5D-3L questionnaire. Moreover the procedure for Informed Consent obtainment has been better detailed.

**Section 5.1. Informed consent pag,12:**

**Original text**

It is the responsibility of the investigator, or a person designated by the investigator, to obtain (if applicable) written informed consent from each individual participating in this study. When applicable, each patient must personally sign and date the latest approved version of the Informed Consent form before any study specific procedures are performed.

**Amended text**

It is the responsibility of the investigator, or a person designated by the investigator, to obtain (if applicable) written informed consent from each individual participating in this study. When applicable, each patient/**health care proxy** must ~~personally~~ sign and date the latest approved version of the Informed Consent form before any study specific procedures are performed.

**Section 5.3.1. Baseline evaluation (CRF B) pag,13:**

**<u>Original text</u>**

[…]

- Self reported Quality of life (EQ-5D-3L). This test will not be administered to patients with moderate severe cognitive impairment (Mini Cog <3)

**<u>Amended text</u>**

[…]

- ~~Self reported~~ Quality of life (EQ-5D-3L, Self or Proxy-1 version). ~~This test will not be administered to patients with moderate severe cognitive impairment (Mini Cog <3)~~
